# Microscopic Origin of Electrochemical Capacitance in Metal–Organic Frameworks

**DOI:** 10.1021/jacs.3c04625

**Published:** 2023-06-21

**Authors:** Seung-Jae Shin, Jamie W. Gittins, Matthias J. Golomb, Alexander C. Forse, Aron Walsh

**Affiliations:** † Department of Materials Science and Engineering, Yonsei University, Seoul 03722, Korea; ‡ Yusuf Hamied Department of Chemistry, University of Cambridge, Cambridge CB2 1EW, U.K.; § Thomas Young Centre and Department of Materials, Imperial College London, London SW7 2AZ, U.K.; ∥ Department of Physics, Ewha Womans University, Seoul 03760, Korea

## Abstract

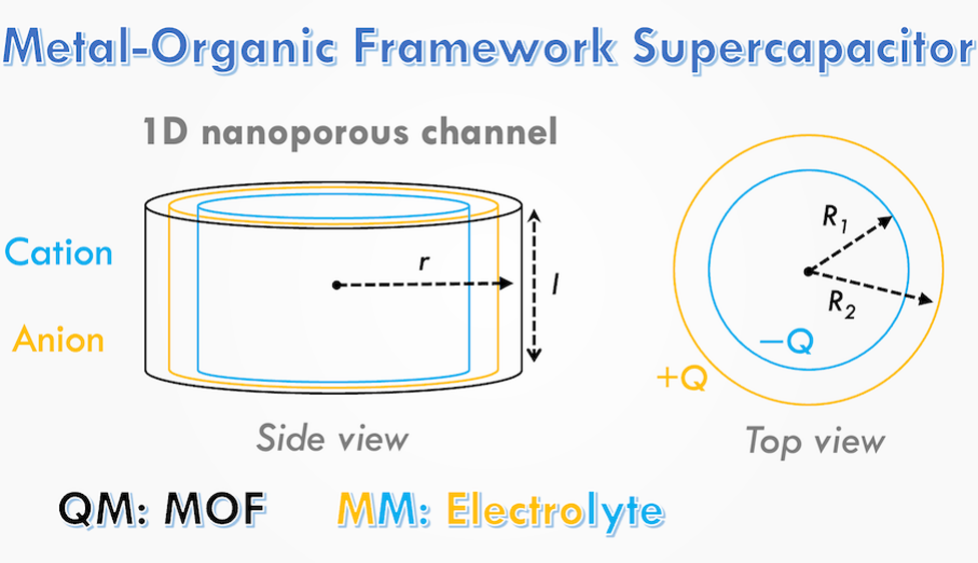

Electroconductive metal–organic frameworks (MOFs) have emerged as high-performance electrode materials for supercapacitors, but the fundamental understanding of the underlying chemical processes is limited. Here, the electrochemical interface of Cu_3_(HHTP)_2_ (HHTP = 2,3,6,7,10,11-hexahydroxytriphenylene) with an organic electrolyte is investigated using a multiscale quantum-mechanics/molecular-mechanics (QM/MM) procedure and experimental electrochemical measurements. Our simulations reproduce the observed capacitance values and reveals the polarization phenomena of the nanoporous framework. We find that excess charges mainly form on the organic ligand, and cation-dominated charging mechanisms give rise to greater capacitance. The spatially confined electric double-layer structure is further manipulated by changing the ligand from HHTP to HITP (HITP = 2,3,6,7,10,11-hexaiminotriphenylene). This minimal change to the electrode framework not only increases the capacitance but also increases the self-diffusion coefficients of in-pore electrolytes. The performance of MOF-based supercapacitors can be systematically controlled by modifying the ligating group.

## Introduction

Supercapacitors are a promising energy-storage technology with high power densities, where charge is stored at the surface of a porous electrode via the formation of an electric double-layer (EDL) upon the application of a voltage.^1^ There have been many efforts to understand their charging mechanisms with porous carbon electrodes, particularly whether the capacitive response originates from counterion insertion, co-ion removal, or ion exchange.^2,3^ For example, in situ electrochemical quartz crystal microbalance (EQCM) and in situ nuclear magnetic resonance spectroscopy (NMR) have been employed to analyze how the stored ionic species depends on the applied potential.^4−6^ Also, molecular dynamics (MD) simulations have been used to observe the interfacial structures at constant charges or constant potentials.^7,8^

Metal–organic frameworks (MOFs) are a new class of electrode materials for energy-storage devices.^9^ In particular, two-dimensional (2D) layered MOFs have exhibited high-performances in supercapacitors because of their relatively high electrical conductivity and outstanding surface area.^10−13^ Furthermore, these materials also have tuneable pore sizes and functional groups,^14^ allowing for systematic modification of the electrode material.

Computational simulations on MOF-based systems have significantly developed in recent years, with studies looking at large-scale screening for gas-separation applications or thermophysical properties^15,16^ to solid–electrolyte interfaces on classical MD simulations.^11^ For electrochemical applications, however, the underlying polarization phenomena are poorly understood, including the role of electrolyte permeation throughout the microporous structure. Although the interfacial structures and charging dynamics at an applied potential have been investigated before,^11^ the direct electronic structure simulation of the electrode incorporating the electrolytes within its pores is challenging due to high computational cost.^17^

Herein, we investigate the charging mechanisms of Cu_3_HHTP_2_ (HHTP = 2,3,6,7,10,11-hexahydroxytriphenylene) incorporating 1 M NEt_4_BF_4_ in acetonitrile electrolyte within the cylindrical pore. We apply a bespoke multiscale quantum-mechanics/molecular-mechanics (QM/MM) technique to supercapacitors for the first time and compare the results with electrochemical experiments on the same system. The QM/MM method can describe the electrode at a QM level with the electrolyte treated classically,^18^ and it has been employed recently to investigate various electrochemical systems with dense metallic electrodes.^19−21^ The treatment of the electrode at the QM level is critical for studying MOFs since they are expected to have heterogeneous electronic structures.^14^ The dynamic structure of the electrolyte is accurately described, including a proper polarization response of the MOF electrode, and the theory-driven capacitance is quantitatively comparable to the experimental measurements with a consistent metal oxidation state. The EDL structure is sensitive to the charging mechanism, and its corresponding potential drop generates charging mechanism-dependent capacitance. By changing the electrode structure, we show that solid–electrolyte interactions can be tuned to modify capacitance, suggesting a design principle for developing MOF-based supercapacitors.

## Results and Discussion

### Electrochemical Interface and Differential Capacitance

**Figure 1:**
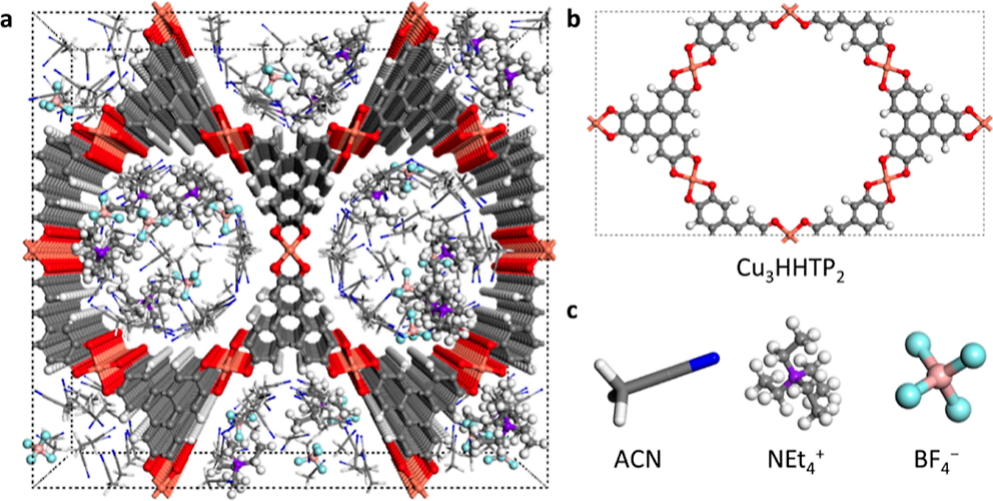
Multiscale QM/MM electrochemical model. (a) Representative QM/MM simulation cell with Cu_3_(HHTP)_2_ electrode and 1 M NEt_4_BF_4_ in acetonitrile electrolyte. (b) Orthorhombic unit cell of Cu_3_(HHTP)_2_ electrode. The atoms are colored to distinguish between C (gray), H (white), O (red), and Cu (orange). (c) Electrolyte components are shown. The B and F atoms are colored pink and cyan, respectively, while the N atoms for acetonitrile and NEt_4_^+^ are colored blue and purple, respectively.

The electrochemical interface comprises the Cu_3_(HHTP)_2_ electrode, a 2D layered MOF which has previously been used as an electrode material in electrochemical applications,^13,22−24^ and 1 M NEt_4_BF_4_ in acetonitrile electrolyte within the cylindrical pore (Supporting Information, Figure S1), a typical organic electrolyte used in supercapacitors (Figure 1).^5,13,25−27^ The QM/MM simulation produces an averaged electrostatic potential profile of the MOF–electrolyte interface at a given surface charge density of the electrode (σ). The potential profiles show plateaus at the pore center region with some fluctuation compared to the profile in vacuum (Supporting Information, Figure S2). Then, the electrode potential (*U*) is calculated from the difference in the plateau potential with the Fermi level,^28,29^ and the potential difference (Δ*U*) from the point of zero charge (PZC) can be readily calculated as a function of σ.

**Figure 2:**
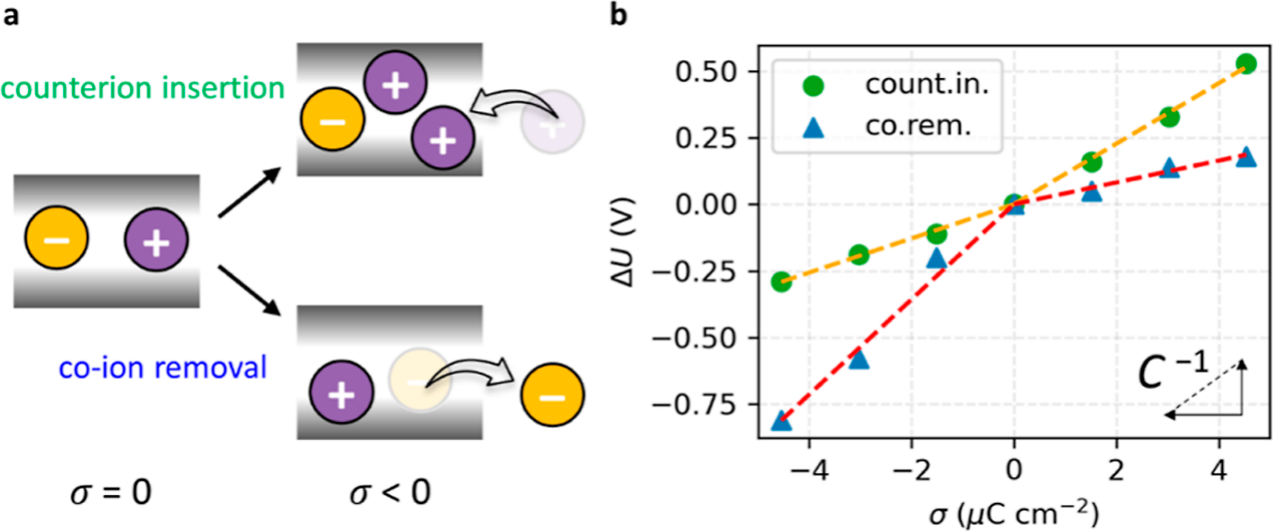
Charging mechanism-dependent differential capacitance. (a) Schematic to show two primary charging mechanisms when the charge density σ < 0. The upper panel shows the counterion insertion (count.in.) mechanism, while the lower panel shows the co-ion removal (co.rem.) mechanism. (b) σ–Δ*U* curve along two different charging mechanisms. The slope indicates the inverse *C*. The *C* values are summarized in Table 1.

There are two primary charging mechanisms considered in this work: (i) counterion insertion and (ii) co-ion removal (Figure 2a), where “co-ion” refers to an ion of which the charge has the same sign as the electrode (e.g., cations for the positive electrode). Ion-exchange (i.e., swapping of counterions and co-ions) will be also discussed to consider all possible charging mechanisms in the experiments.^2,30^ The ion-exchange ratio is quantified using the charging mechanism parameter,^2^*X*(σ) parameter, defined as
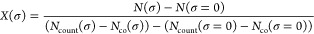
where *N*(σ), *N*_count_(σ), and *N*_co_(σ) are the number of in-pore total ions, counterions, and co-ions, respectively, at a certain σ. *X* = 1 for counter-ion insertion, *X* = 0 for ion-exchange, and *X* = −1 for co-ion removal, with intermediate values of *X* also possible.

Δ*U* shows a charging mechanism-dependent trend when the σ is controlled from −4.5 to +4.5 μC cm^–2^, where the counterion insertion mechanism causes Δ*U* to vary from −0.3 to 0.5 V, whereas the co-ion removal causes Δ*U* to vary over a greater range from −0.8 to 0.2 V (Figure 2b). The Δ*U* from −1 < *X* < 1 is located in between *X* = −1 (co-ion removal) and *X* = 1 (counterion insertion) at each polarized electrode (Supporting Information, Figure S3), calculated from the electrostatic potential profiles (Supporting Information, Figure S4). Then, the differential capacitance (*C*) is calculated based on its definition, i.e., *C* = σ/Δ*U*, and it ranges from 8 to 24 μF cm^–2^ for 0 < σ < +4.5 μC cm^–2^ and 6 to 18 μF cm^–2^ for −4.5 < σ < 0 μC cm^–2^, depending on the charging mechanism (Table 1). Summarizing, for positive charging, the co-ion removal mechanism is calculated to give a higher capacitance, whereas, for negative charging, the counterion insertion mechanism gives a higher capacitance. For both σ ranges, a higher capacitance is predicted when the cations are the primary charge carrier. The origin of charging mechanism-dependent capacitance will be discussed later.

**Table 1:** Differential Capacitance from QM/MM Simulations of the Cu_3_(HHTP)_2_ Electrochemical Interface^a^

QM/MM simulation		capacitance (μF cm^–^^2^)	
*X* value	charging mechanism	–4.5 < σ < 0 μC cm^–^^2^	0 < σ < +4.5 μC cm^–^^2^
1.0	counterion insertion	18	8
0.5		12	12
0.0	ion-exchange	8	12
–0.5		7	11
–1.0	co-ion removal	6	24

experiment	capacitance (μF cm^–^^2^)		
potential/V *vs.* OCV	negative	positive	switching
0.5^b^	14.9	17.7	18.9

a The capacitance is calculated to read the inverse value of the slope in Figure 2b and Supporting Information, Figure S3. The experimental range is calculated from galvanostatic charge–discharge curves by charging to positive potentials *vs*. OCV, to negative potentials *vs*. OCV, and between both positive and negative potentials of the same magnitude denoted as “Switching”. “Positive”, “Negative”, and “Switching” means the potential ranges from 0.5 to 0 V, −0.5 to 0 V, and +0.5 to −0.5 V *vs*. OCV, respectively. The data were collected between ±0.5 V *vs*. OCV, which is the stable potential window for this MOF and at a current density 0.05 A g^–1^ to limit kinetic effects. Gravimetric capacitance values are summarized in Supporting Information, Table S2.

b Average of two independent measurements.

Cu_3_(HHTP)_2_ was synthesized and characterized with X-ray diffraction (XRD) (Supporting Information, Figure S5) and Brunauer–Emmett–Teller (BET) analyses (Supporting Information, Figure S6). Three-electrode electrochemical experiments were carried out with the same system for comparison with the QM/MM calculations (Supporting Information, Figure S7). Experimental areal capacitance values were obtained from galvanostatic charge–discharge experiments at a current density of 0.05 A g^–1^ (Supporting Information, Figures S8 and Table S1). Values were obtained for charging to both the positive and negative stable potential limits relative to the open circuit voltage (OCV) of the cell (+0.5 and −0.5 V *vs*. OCV, respectively). From these experiments, we report areal capacitance values of 16.9–18 μF cm^–2^ for positive charging and 13.8–16.0 μF cm^–2^ for negative charging. These results are similar to the previous two electrode measurements, which gave capacitance values in the range of 14–23 μF cm^–2^.^13^

The experimental areal capacitance values fall within the range of simulated values (Table 1), supporting that our novel QM/MM method is a good model for this system and gives reliable capacitance values close to the experimental values. Interestingly, the simulated and experimental areal capacitance values most closely match when the value of *X* is approaching −1 for positive charging and +1 for negative charging. For both of these *X* values, the cations are the primary charge carriers. Therefore, this work suggests that the experimental charging mechanism for Cu_3_(HHTP)_2_ with 1 M NEt_4_BF_4_ in acetonitrile electrolyte may be dominated by the movement of cations. To the best of our knowledge, this is the first computational prediction of the charging mechanism of 2D layered MOF-based supercapacitor, and further experimental work is needed to confirm this exciting prediction.

Further support for our simulation method is obtained from calculations on a graphite surface (Supporting Information, Figure S9), which give consistent results with previous reports.^31,32^ Moreover, this approach can capture the subtle interactions between MM-described electrolyte molecules and QM-described reactants or electrode surface at the electrochemical interface.^19−21^ Thus, it is convincing that the QM/MM method can describe the realistic interfacial structure with the porous electrode, and it provides theoretical upper and lower bounds for the capacitance, depending on the charging mechanisms.

### Polarization Phenomena of MOFs

The QM/MM approach allows the electronic structure of the MOFs to be modeled quantum mechanically, in contrast to classical MD simulations,^11^ providing a unique opportunity to understand polarization phenomena. To study the electronic polarization of the Cu_3_(HHTP)_2_ electrode interface during charging with different charging mechanisms, the system was set to a target potential of ±0.2 V *vs.* PZC. The target potentials were chosen to clearly see the charging mechanism-dependent behavior based on the constant capacitance up to these potentials from the PZC for each charging mechanism in our σ–Δ*U* curve (Figure 2b).

**Figure 3:**
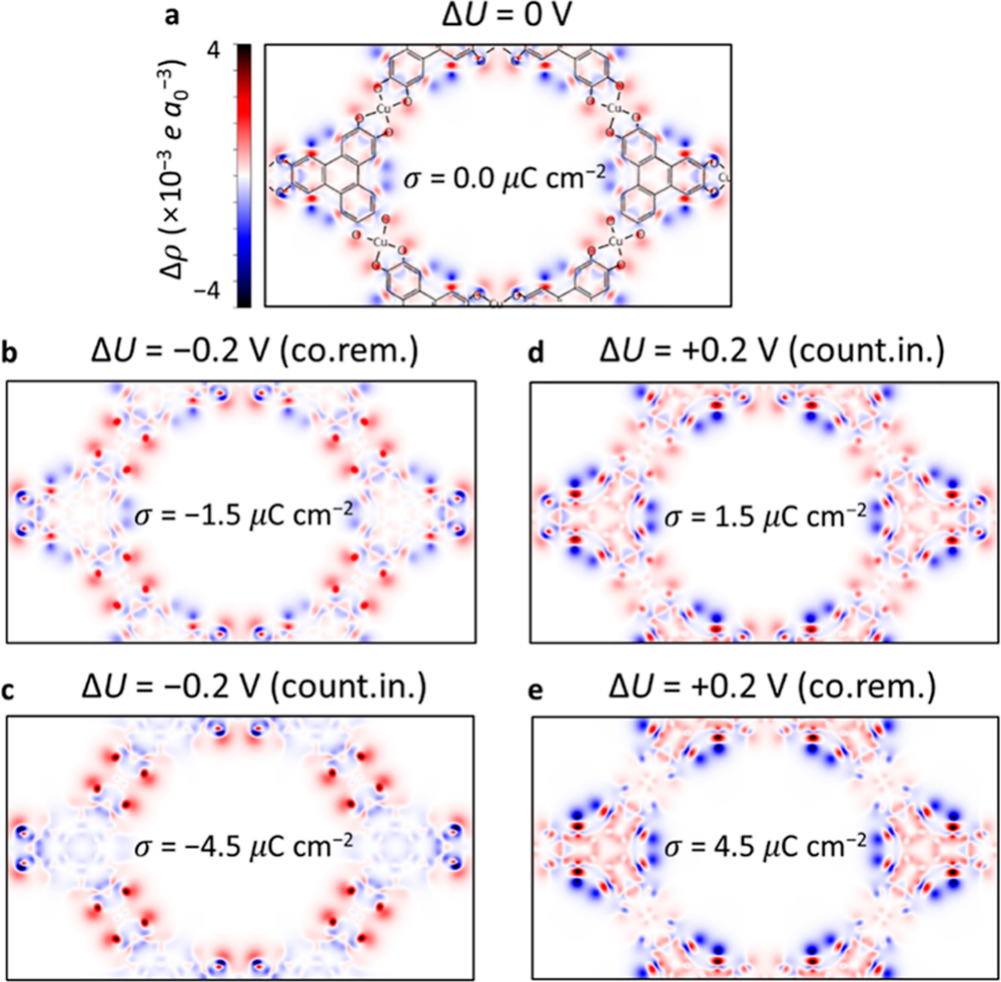
Electronic polarization of Cu_3_(HHTP)_2_ at the electrochemical interface. (a) Charge density difference (Δρ) referring to the vacuum condition is shown when σ is 0.0 μC cm^–2^. The accumulated or depleted electron density is colored red or blue, respectively. The following figures all share the same color code. The electrolyte molecules are omitted to provide a clear view. (b,c) When Δ*U* is −0.2 V, Δρ referring to the PZC is shown following the co-ion removal (co.rem.) mechanism (b) and counterion insertion (count.in.) mechanism (c). (d,e) When Δ*U* is +0.2 V, Δρ referring to the PZC is shown following the count.in. mechanism (d) and co.rem. mechanism (e).

Prior to charging, the electrode becomes more polarized after interacting with the electrolyte compared to the vacuum condition, with the main charge density changes on the HHTP organic linker showing more accumulated electron density on the O atoms and depleted electron density on the H atoms (Figure 3a). After charging the electrode, the polarization phenomena are dependent on the charging mechanism. When Δ*U* is calculated as −0.2 V, the co-ion removal mechanism corresponds to the electrode having a σ of −1.5 μC cm^–2^ (Figure 3b), while the counterion insertion mechanism has a σ of −4.5 μC cm^–2^ which is three times higher (Figure 3c). On the other hand, when Δ*U* is measured as +0.2 V, the trend is reversed. The electrode has a σ of +1.5 μC cm^–2^ with the counterion insertion mechanism (Figure 3d), but the co-ion removal mechanism leads to a σ of +4.5 μC cm^–2^ which is greater by a factor of 3 (Figure 3e). For both Δ*U*, the electrode is more polarized when the cations are the primary charge carriers, and its origin will be discussed in the following section.

**Figure 4:**
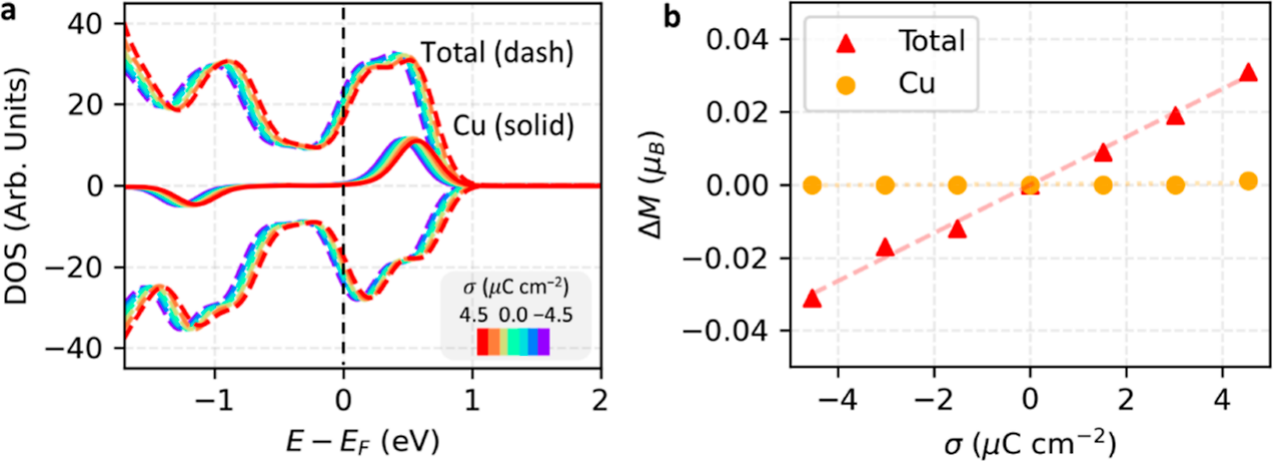
Electronic structure of Cu_3_(HHTP)_2_ at the electrochemical interface. (a) DOS near the Fermi level is shown at each σ. The total DOS is plotted with the dashed line, while the partial DOS of Cu is plotted with the solid line. The simulation cell has an anti-ferromagnetic spin configuration, and only one configuration is shown for the Cu atom. (b) Difference of magnetic moment (Δ*M*) referring to the PZC with respect to σ. The total Δ*M* is per simulation cell. All figures are constructed following the counterion insertion. Supporting Information, Figure S10 shows a similar behavior following the co-ion removal mechanism.

During charging or discharging, the excess charges remain localized to the organic linker, and Cu shows only a marginal charge density change, independent of the charging mechanism. The electronic density of states (DOS) shows that Cu has a 3d^9^ open-shell doublet electronic configuration and is thus in the 2+ oxidation state following the counterion insertion mechanism (Figure 4a) and the co-ion removal mechanism (Supporting Information, Figure S10a). The DOS is largely unchanged with the Fermi level, indicating weak electronic coupling, and the Cu atom maintains a magnetic moment of 0.6 μ_B_ in this potential window, comparable to the experimental measurements and previous computational studies.^33−37^ The total magnetization of the Cu_3_(HHTP)_2_ simulation cell increases by 0.05 μ_B_ from the PZC, supporting the localization of excess charges on the organic moieties for both charging mechanisms (Figure 4b and Supporting Information, Figure S10b). X-ray absorption near edge structure (XANES) measurements support the persistence of Cu^2+^ with no Faradaic reactions observed in the experimental potential window.^13^

### Origin of the Charging Mechanism Dependent Capacitive Behavior

**Figure 5:**
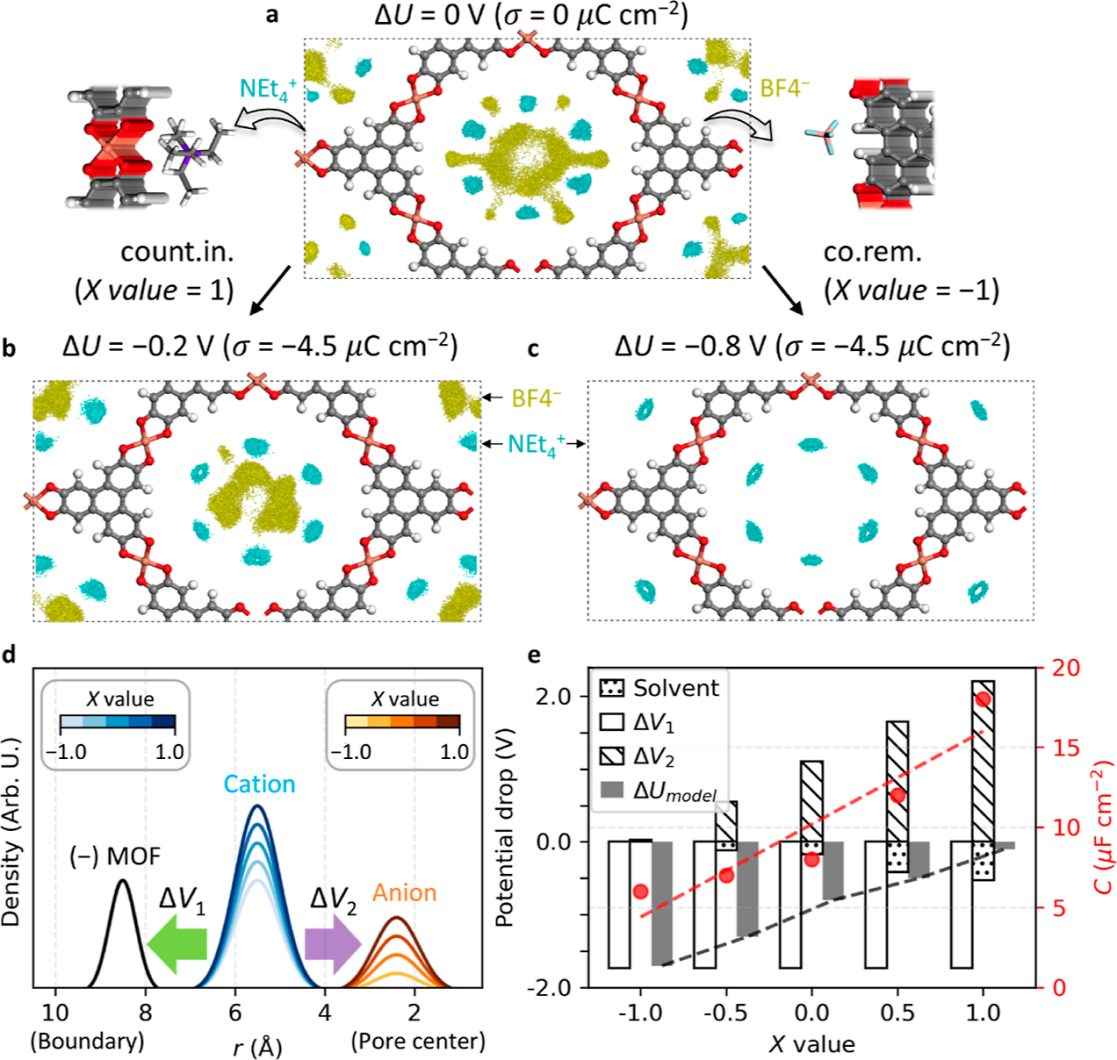
Charging mechanism-dependent EDL structure. (a–c) Isosurface of the time-averaged ion distribution is shown at the PZC (a), and σ is −4.5 μC cm^–2^ following the counterion insertion (count.in.) mechanism (b) and co-ion removal (co.rem.) mechanism (c). The distribution of cations or anions is colored cyan or yellow, respectively, using their center of mass. The isosurface level is 0.001 e bohr^–3^. The acetonitrile distribution is omitted to provide a clear view. The adsorption site of each ion to the electrode at the PZC is shown in the inset. (d) Density of excess charge is illustrated with respect to the radial distance (*r*) from the center of the MOFs, when σ is −4.5 μC cm ^–2^. It is based on the Supporting Information, Figure S19. The saturation of color indicates the density of components at each *X* value. The two major electric fields between the different components are shown with colored arrows named Δ*V*_1_ and Δ*V*_2_. (e) Potential drop inside the MOFs is shown with respect to the *X* value using cylindrical capacitor models. The total potential drop (Δ*U*_model_) is decomposed into three terms, where two of them are Δ*V*_1_ and Δ*V*_2_. The potential drop due to the effective dielectric screening from the solvent molecules (solvent) is also shown. *C* value from the QM/MM simulation is plotted together.

The EDL structure can be decomposed into two regions: (i) the pore boundary and (ii) the pore center. At the PZC, all NEt_4_^+^ adsorb at Cu–O_4_ sites while BF_4_^–^ are partitioned between adsorption at the C–H units of the organic linker and the pore center (Supporting Information, Figure S11a). The cation or anion loses 2 or 3 coordinated solvent molecules, respectively, when adsorbing to the electrode (Supporting Information, Figure S12). This electrolyte structuring results from specific electrostatic interactions with the electrode (Supporting Information, Figure S13). A heterogeneous ion distribution is induced at the pore boundary region with a relatively homogenous anion distribution at the pore center region (Figure 5a).

When the electrode is negatively polarized to a σ value of −4.5 μC cm^–2^, the NEt_4_^+^ remain at the boundary region independent of the charging mechanism, while BF_4_^–^ are mainly located at the pore center region, with a loss of the population at the pore boundary region (Figure 5b,c and Supporting Information, Figure S11b,c). When the electrode is positively polarized to a σ value of +4.5 μC cm^–2^, almost all BF_4_^–^ exist at the pore boundary region with the co-ion removal mechanism, but unignorable amounts are accumulated at the pore center region following the counterion insertion mechanism (Supporting Information, Figures S11d,e and 14), owing to the interaction with the existing NEt_4_^+^ (Supporting Information, Figure S15). Our observation of a heterogeneous ion distribution parallels that seen in a previous MD study with MOF–ionic liquid interfaces,^11^ suggesting that this might be a general response of the EDL in the cylindrical pores of MOFs. It originates from the inhomogeneous but regularly arranged functional groups around the boundary region, strikingly different from porous carbon electrodes. The functional groups then interact more favorably with certain electrolyte species due to differences in binding energies, and the resultant localization of ions has a critical role in determining the capacitance.

The in-pore solvent can also affect the capacitance.^19,38−40^ Density profiles and radial distribution of solvent molecules from the electrode shows that the acetonitrile molecules at the boundary region are aligned such that the N-head or alkyl group is coordinated to the organic compound or Cu–O_4_ fragment of the MOF, respectively, contributing to the polarized electronic structure of the MOF at the PZC (Supporting Information, Figures S16 and 17). During the charging and discharging, they rotate to screen the σ, where their dipolar orientation to the surface changes systematically (Supporting Information, Figure S18), and their contributions to the capacitance are quantified in Supporting Information, Note S1.

The charging mechanism changes the in-pore EDL structure. This results in a characteristic capacitance and determines the electrode potential at a given σ. The charging mechanism-dependent capacitance can be explained from fundamental electrostatics using a cylindrical capacitor model (Supporting Information, Note S2). The density of ions increases as the *X* parameter increases, where two potential drops occur (Figure 5d and Supporting Information, Figure S19). The first drop, Δ*V*_1_, is between the excess electrons in the MOF, and the cations at the boundary region. It is almost independent of the charging mechanism. The second drop, Δ*V*_2_, is formed between cations at the boundary and anions at the pore center and has the opposite sign to Δ*V*_1_. As *X* increases, Δ*V*_2_ changes from 0 to 2.2 V. On the other hand, the dielectric screening from the solvent changes from 0 to −0.5 V, ascribed to the increased ion density and the reduced number of in-pore solvent molecules in the confined space (Supporting Information, Note S1).^19,41^ Overall, the magnitude of the total potential drop, Δ*U*_model_ changes from 1.6 to 0.1 V. Higher capacitance is achievable with the large *X* because the greater ion density can induce a smaller potential drop for the same σ.

Based on the quantitative comparison of the capacitance, the experimental charging mechanism is predicted above as dominated by the movement of cations. However, experimental measurements of the charging mechanism should be carried out in the future (e.g., using in situ EQCM or in situ NMR) to further investigate the charging mechanism of MOF-based supercapacitors. An interesting lesson from the QM/MM simulations is that higher capacitance is generated when NEt_4_^+^ plays a major role in charging because of its strong interaction with the Cu_3_(HHTP)_2_ electrode, i.e., counterion insertion at a negatively charged electrode and co-ion removal at a positively charged electrode. We note that there have been attempts to achieve higher capacitance by controlling the charging mechanism.^27,42−44^

### Modulating the EDL Structure with MOF Composition

The EDL structure can be modulated by taking advantage of the chemical tuneability of MOFs.^45^ We therefore explored the electrochemical interface of a second 2D layered MOF, Cu_3_(HITP)_2_ (HITP = 2,3,6,7,10,11-hexaiminotriphenylene), which has a similar pore size to Cu_3_(HHTP)_2_ (Supporting Information, Figure S20).^46^ The electrode polarization shows that the excess charge is once again localized on the organic ligand, and the oxidation state of Cu is maintained as 2+, independent of the biased potential, and consistent with our above results for Cu_3_(HHTP)_2_ (Supporting Information, Figure S21).

**Figure 6:**
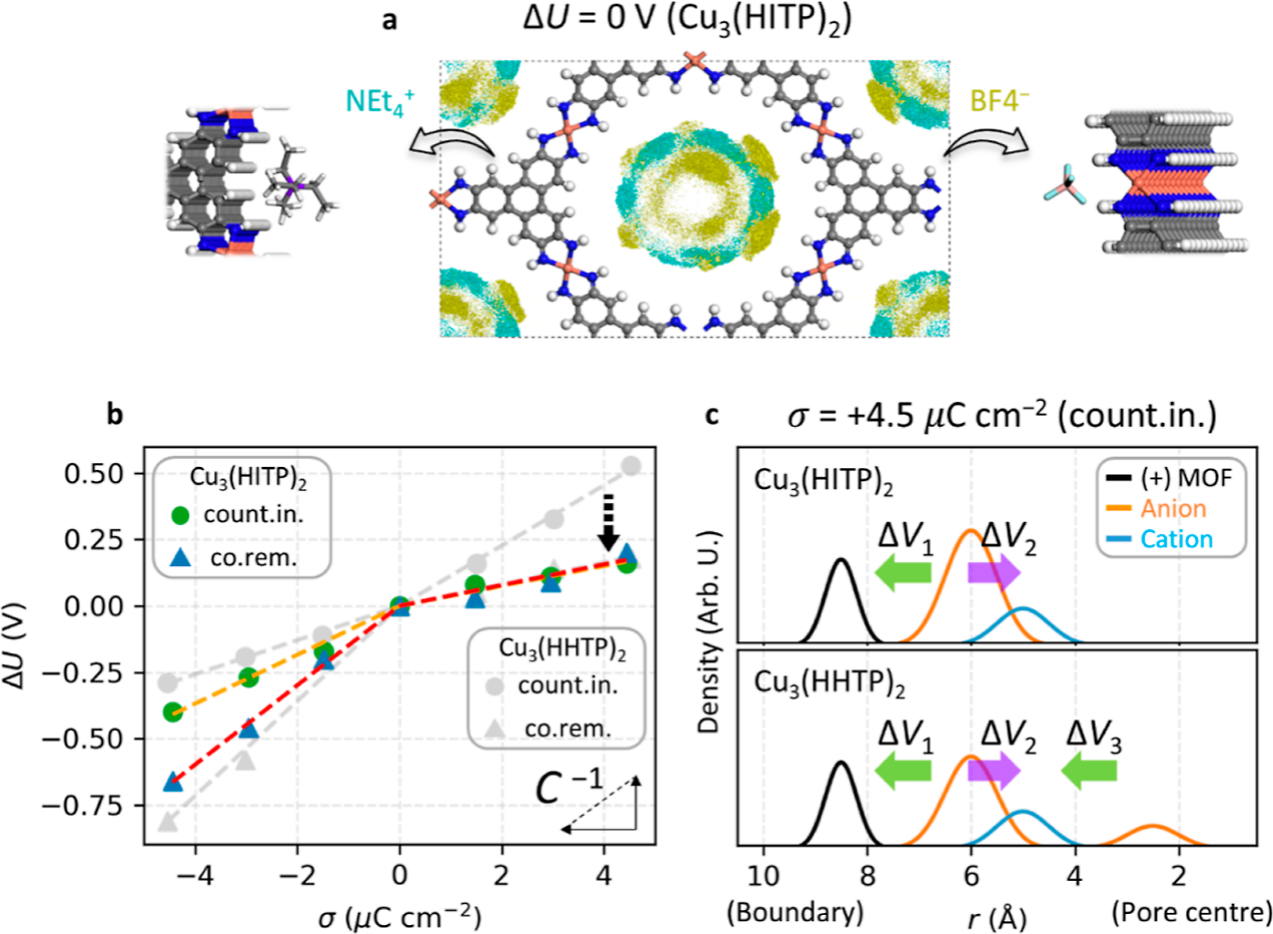
EDL structure modulation and differential capacitance control. (a) Isosurface of the time-averaged ion distribution at PZC with the Cu_3_(HITP)_2_ electrode. The N atom for HITP is colored blue. The distribution of cations or anions is colored cyan or yellow, respectively using their center of mass. The isosurface level is consistent for both ones as 0.001 e bohr^–3^. The adsorption site of each ion to the electrode at the PZC is shown in the inset. (b) σ–Δ*U* curve to compare the capacitance from Cu_3_(HHTP)_2_ and Cu_3_(HITP)_2_. The slope indicates the inverse *C*. The *C* values are summarized in Table 2. (c) Density of excess charge is illustrated with respect to the radial distance (*r*) from the center of the MOFs, when σ is +4.5 μC cm^–2^ following the counterion insertion (count.in.) mechanism. It is based on the Supporting Information, Figures S19 and 31. The two or three electric fields between the different components are shown with colored arrows named Δ*V*_1_, Δ*V*_2_, and Δ*V*_3_.

However, the EDL structure of Cu_3_(HITP)_2_ is different from that of Cu_3_(HHTP)_2_ at the PZC because of different interactions between the 2D MOF and electrolyte components (Supporting Information, Figure S22). The NEt_4_^+^ are now distributed quite homogeneously at the boundary region, and the BF_4_^–^ prefer to bind to the Cu-(NH)_4_ units, which were absent in Cu_3_(HHTP)_2_ (Figure 6a). Density profiles and radial distribution of solvent molecules from the electrode shows that the acetonitrile molecules at the boundary region are now aligned with the N-head coordinated to the N–H groups on the organic ligand (Supporting Information, Figures S23 and 24), and it shows a similar screening property during charging to the Cu_3_(HHTP)_2_ system (Supporting Information, Figure S25). Notably, the most critical change compared to Cu_3_(HHTP)_2_ is observed when σ is +4.5 μC cm^–2^, where the capacitance following the counterion insertion mechanism dramatically increases (Figure 6b), calculated from the electrostatic potential profiles (Supporting Information, Figure S26). It is ascribed to the distribution of the anions which nearly disappears at the pore center region, a stark contrast to the Cu_3_(HHTP)_2_ system (Supporting Information, Figures S27 and 28). This minimizes the potential drop between the cations at the boundary region and anions at the pore center region, Δ*V*_3_, resulting in a higher capacitance (Figure 6c). Therefore, a higher averaged capacitance is also obtained by employing Cu_3_(HITP)_2_ as the electrode material compared to Cu_3_(HHTP)_2_ (Supporting Information, Figure S29 and Table 2), calculated from the electrostatic potential profiles in all range of *X* value (Supporting Information, Figure S30). The cylindrical capacitor model also helps explain the capacitance change using the Cu_3_(HITP)_2_ electrode (Supporting Information, Note S2), based on its in-pore ion distribution (Supporting Information, Figure S31).

**Table 2:** Differential Capacitance from the QM/MM Simulation of the Cu_3_(HITP)_2_ Electrochemical Interface^a^

		capacitance (μF cm^–^^2^)	
X value	charging mechanism	–4.5 < σ < 0 μC cm^–^^2^	0 < σ < +4.5 μC cm^–^^2^
1.0	counterion insertion	17	25
0.5		13	24
0.0	ion-exchange	11	23
–0.5		10	23
–1.0	co-ion removal	9	25

a The capacitance is calculated to read the inverse value of the slope in Figure 6b and Supporting Information, Figure S29.

The modified interfacial interaction also greatly increases the self-diffusion coefficients of all components of the electrolyte at the PZC (Supporting Information, Figure S32), compared to those in Cu_3_(HHTP)_2_ (Supporting Information, Figure S33). Cu_3_(HHTP)_2_ shows an over 70% decreased self-diffusion coefficient compared to the bulk electrolyte^32^ due to the confinement effect in the pores and interactions with the electrode material.^47,48^ This is consistent with previous experimental measurements on different MOF-electrolyte systems.^49,50^ The self-diffusion coefficient parallel to the hexagonal pores is two times larger than that perpendicular to the pores, indicating the anisotropic nature of the self-diffusion of the in-pore electrolyte. Thus, Cu_3_(HITP)_2_ also has kinetic benefits for supercapacitor systems based on the fast movement of ions inside the pores.^2^

Superior MOF-based supercapacitors could be developed based on these theoretical principles in the future. However, additional factors affect their performance, including the electrode morphology,^51^ which may hinder the practical application of certain electrode–electrolyte combinations. Nevertheless, these findings show that the functional groups in the ligands of MOFs can significantly impact both the EDL structure and the resulting capacitance values, despite similar pore structures and pore sizes. This suggests a new approach for developing future hybrid supercapacitors.

## Conclusions

In summary, we have deciphered the complex electrode–electrolyte interfacial structure of a MOF-based supercapacitor system using multiscale QM/MM electrochemical simulations, supported by direct measurements of the same system. The QM/MM simulations make it possible to model and study the polarization phenomena of MOFs at the electrochemical interface. The charging mechanism controls the capacitance by changing the characteristic EDL structure of the system, with cation-dominated charging mechanisms giving rise to higher capacitances with tetraethylammonium-based electrolytes. A higher supercapacitor-performance is also predicted from the QM/MM simulation by changing the organic ligand while maintaining the porous architecture. It induces an increased capacitance and a higher self-diffusion coefficient of the organic electrolyte, originating from the modulated EDL structure. Thus, this work provides a design principle to develop improved MOF-based supercapacitors by highlighting the importance of the confined solid-liquid interfacial structure.

## Materials and Methods

### Computational Details

The mean-field QM/MM multiscale electrochemical simulation, namely, density functional theory in classical explicit solvents (DFT-CES),^18^ is implemented in a bespoke code that combines the Quantum ESPRESSO and LAMMPS.^52,53^ The DFT-CES iteration was repeated until the difference of the DFT total energy between the iterations converged below 0.1 kcal mol^–1^. At every iteration, 7 ns MD simulation was performed, and the last 5 ns trajectory was sampled to average the electrostatic potential of the electrolyte phase that was employed in the subsequent DFT calculation as an external potential.

The Cu_3_(HHTP)_2_ electrode was quantum-mechanically modeled using two layers with a () rect. unit cell with dimensions of 20.4 Å × 37.9 Å × 7.24 Å. The electrode has an inclining-layered structure with a constant stacking shift based on a previous study.^13^ The Perdew–Burke–Ernzerhof (PBE) exchange–correlation functional with dispersion correction via Grimme’s scheme (DFT + D3) was employed.^54,55^ For an accurate description of the localized *d* electrons of Cu, the on-site Coulomb interaction was added to the *d* orbital of Cu with a *U*_*d*_ = 4.0 eV.^56,57^ The projector-augmented-wave (PAW) method was used with a kinetic energy cutoff of 50 Ry with charged density cutoff of 500 Ry,^58^ and the Gaussian smearing was used with a value of 0.2 eV. A (2 × 2 × 2) Γ-centered *k*-point grid was used to sample reciprocal space. These parameters produce a voxel grid of volume 0.073 Å^3^, satisfying the suggested value of 0.13 Å^3^. The Cu_3_(HITP)_2_ electrode was modeled with a () rect. unit cell with the dimensions of 20.93 Å × 38.65 Å × 3.60 Å. All parameters were the same as those of the Cu_3_(HHTP)_2_ electrode, except for the *k*-point grid which was a Γ-centered (2 × 2 × 4) grid. This was done to compensate for the halved size of the supercell, which was necessary due to technical problems related to computational memory size.

The electrolyte was classically modeled using the canonical ensemble MD. The one-dimensional hexagonal pores in the MOFs were filled with 1 M NEt_4_BF_4_ in acetonitrile, assuming that the density of the electrolyte is consistent with that of the bulk electrolyte. The free volume of the electrode was estimated using the Connolly surface area by employing a probe molecule with a kinetic diameter of 3.68 Å, equivalent to the kinetic diameter of N_2_. Nosé–Hoover thermostat was employed to set the temperature at 300 K,^59,60^ with a damping parameter of 100 fs. The OPLS-AA force field (FF) was employed to describe the interatomic potential,^61^ as, based on a previous paper, the molecular solvation energy can be descried accurately from the DFT-CES with OPLS-AA FF.^18^ For the Cu atom, the van der Waals (vdW) FF parameters for the Lennard-Jones (LJ) potential were carefully developed to accurately describe the interfacial interactions. To determine the parameters, we obtained the binding energy curves between the electrolyte components and a fragment of Cu_3_(HHTP)_2_ monolayer, comprising one Cu atom, four O atoms, and 2 benzene molecules (Supporting Information, Figure S13). The parameters of the Cu atom in Cu_3_(HITP)_2_ were also obtained in a similar manner (Supporting Information, Figure S22). The B3LYP-D3 functional with the LACVP**++ basis set, consisting of the LANL2DZ effective core basis set for Cu, and standard Pople’s 6-31G**++ basis set for other elements,^62^ using the NWChem software.^63^ The external potential from the electrode in the QM region was set as follows: the DFT optimized structure and electrostatic potential obtained from the () rect. model was repeated to fill the () rect. model, resulting in an MD simulation cell dimension of 40.8 Å × 37.9 Å × 43.4 Å.

### Material Synthesis

All chemicals were purchased from commercial suppliers and used without modification unless stated.

A previously published procedure was used to synthesize Cu_3_(HHTP)_2_.^51^ In brief, a solution of Cu(NO_3_)_2_·3H_2_O (0.127 g, 0.526 mmol, 1.65 eq) and aqueous ammonia (35%, 0.883 mL, 50 equiv) in distilled water (2 mL) was prepared. The resulting royal blue solution was added dropwise to a dispersion of HHTP (0.103 g, 0.318 mmol, 1.00 equiv) in distilled water (8.2 mL). The resulting mixture was heated in a furnace oven at 80 °C for 24 h. The dark blue precipitate formed was separated by centrifugation. The precipitate was then washed successively with water (3 × 30 mL), ethanol (3 × 30 mL), and acetone (3 × 30 mL). The precipitate was then filtered by vacuum filtration, and the resulting dark blue powder was dried at 80 °C under dynamic vacuum for 96 h and then stored in a N_2_-filled glovebox until used.

Freestanding composite Cu_3_(HHTP)_2_ films were prepared using an existing literature method.^13^ In brief, the electroactive components were ground together in a vial before ethanol (*ca.* 1.5 mL) was added to produce a loose slurry. This was sonicated for 15 min before being added to PTFE dispersion (60 wt % in water) in a few drops of ethanol. The slurry was stirred by hand for 40 min under ambient conditions. The film was formed upon drying of the slurry and was kneaded for 20 min to ensure homogeneity before being rolled into a freestanding electrode film using a homemade aluminum rolling pin. The film was dried in vacuo at 100 °C for at least 48 h to remove any remaining ethanol. The masses of components were calculated so that the final films had a composition of 85 wt % Cu_3_(HHTP)_2_, 10 wt % acetylene black (measured BET area = 62 m^2^ g^–1^), and 5 wt % PTFE. All films had a thickness of *ca*. 250 μm. YP80F films were made using the same method.

### Materials Characterization

High-resolution synchrotron XRD data were collected at the I11 beamline at Diamond Light Source. Samples were loaded into borosilicate glass capillary tubes (0.5 mm outside diameter, 0.01 mm wall thickness; Capillary Tube Supplies Ltd.) in a N_2_-filled glovebox, and then sealed with Loctite EA 3430 epoxy adhesive. Diffraction patterns were collected under ambient conditions using a Mythen II position-sensitive detector (PSD) with two 5 s scans separated by an angular shift in the detector position of 2.5°. The wavelength and intrinsic peak–shape parameters were refined against a known Si 640c NIST standard. The refined wavelength for the PSD scans was 0.82683 Å (∼15 keV). Simulated XRD patterns were produced using VESTA version.^64^

Low-pressure N_2_ isotherms (adsorption and desorption) were collected using an Anton Parr Autosorb iQ-XR at 77 K. Ex situ degassing (80 °C, 24 h) was performed and isotherms were collected over 24–30 h. Sorption isotherms were evaluated in AsiQwin version 5.21 software. Material BET areas were calculated from isotherms using the BET equation and Rouquerol’s consistency criteria implemented in AsiQwin.^65,66^

### Electrochemical Measurements

Three-electrode cells were prepared in Swagelok PFA-820-3 union tube fittings with homemade stainless-steel plugs as current collectors. Cu_3_(HHTP)_2_ composite electrodes with areal mass loadings ranging between 11 and 12 mg cm^–2^ were used as working electrodes. Overcapacitive YP50F activated carbon film electrodes with areal mass loadings of 35–40 mg cm^–2^ were used as counter electrodes. Ag wire was used as a pseudo-reference electrode. A 1 M solution of NEt_4_BF_4_ in anhydrous acetonitrile was used as the electrolyte. Before being transferred to the glovebox, NEt_4_BF_4_ was dried at 100 °C under vacuum for 4 days and anhydrous acetonitrile was purged with nitrogen for 3 h. The amount of electrolyte added was controlled at 750 μL. Whatman glass microfiber filter (GF/A) was used as a separator, and two separators were added to each cell. The cells were hermetically sealed by hand and removed from the glovebox for testing. Under these conditions, the ferrocene–ferricenium (Fc/Fc^+^) redox couple was measured at 0.564 ± 0.002 V *vs* Ag. All potentials discussed for the three-electrode cell are referenced to Ag.

All electrochemical measurements were carried out using a Biologic VSP-3e potentiostat. The areal capacitance is determined by normalizing a measured gravimetric capacitance with a measured surface area of Cu_3_(HHTP)_2_ in an electrode film from gas sorption measurements. All experimental capacitance values for Cu_3_(HHTP)_2_ were calculated after removing the contributions from acetylene black and PTFE that are also present in the electrodes.

## Supporting Information Available

Computational setup, experimental characterization, additional electrochemical analysis (PDF)

## Notes

All data is available in the main text or Supporting Information. The main data has been uploaded to the Zenodo repository at https://doi.org/10.5281/zenodo.7981421. The DFT-CES code used in this work has been deposited in a Github repository with the permanent link https://doi.org/10.5281/zenodo.7037865.^67^
