# Supplementary material for: Microscopic Origin of Electrochemical Capacitance in Metal–Organic Frameworks
Source: J Am Chem Soc. 2023 Jun 21;145(26):14529–38. doi: 10.1021/jacs.3c04625 (PMC10326873; doi:10.1021/jacs.3c04625)
Supplement: Supplementary file 1 — ja3c04625_si_001.pdf [file ja3c04625_si_001.pdf]

Supplementary Information for

Microscopic Origin of Electrochemical Capacitance in Metal-Organic Frameworks

Authors: Seung-Jae Shin[1], Jamie W. Gittins[2], Matthias J. Golomb[3], Alexander C. Forse[2*], and Aron Walsh[3,4*]

Affiliations:

[1]Department of Materials Science and Engineering, Yonsei University, Seoul 03722, Korea

[2]Yusuf Hamied Department of Chemistry, University of Cambridge, Cambridge CB2 1EW, UK

[3]Thomas Young Centre and Department of Materials, Imperial College London, London SW7 2AZ, UK

[4]Department of Physics, Ewha Womans University, Seoul 03760, Korea

*acf50@cam.ac.uk and a.walsh@imperial.ac.uk

This PDF file includes:

Supplementary Figure 1 to 33

Supplementary Table 1 to 2

Supplementary Note 1 to 2

Supplementary Reference

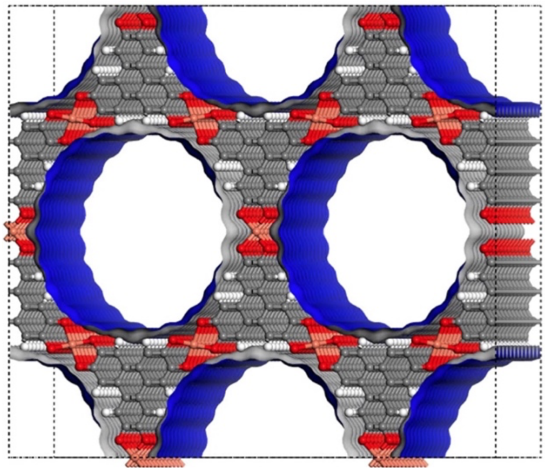

Supplementary Fig. 1. Surface area of the $Cu_3(HHTP)_2$ simulation cell used for molecular dynamics. The surface area is estimated as 1286 m^2 g^{-1} by the Connolly surface area employing a probe molecule with a kinetic diameter of 3.68 Å, equivalent to the kinetic diameter of N_2. The organic electrolyte fills the pores of the electrode. The number of electrolyte molecules is controlled to compensate the electrode charge. For cation insertion, three acetonitrile molecules are ejected from the pore, while anion insertion results in the removal of one acetonitrile molecule from the pore based on their occupied volume.

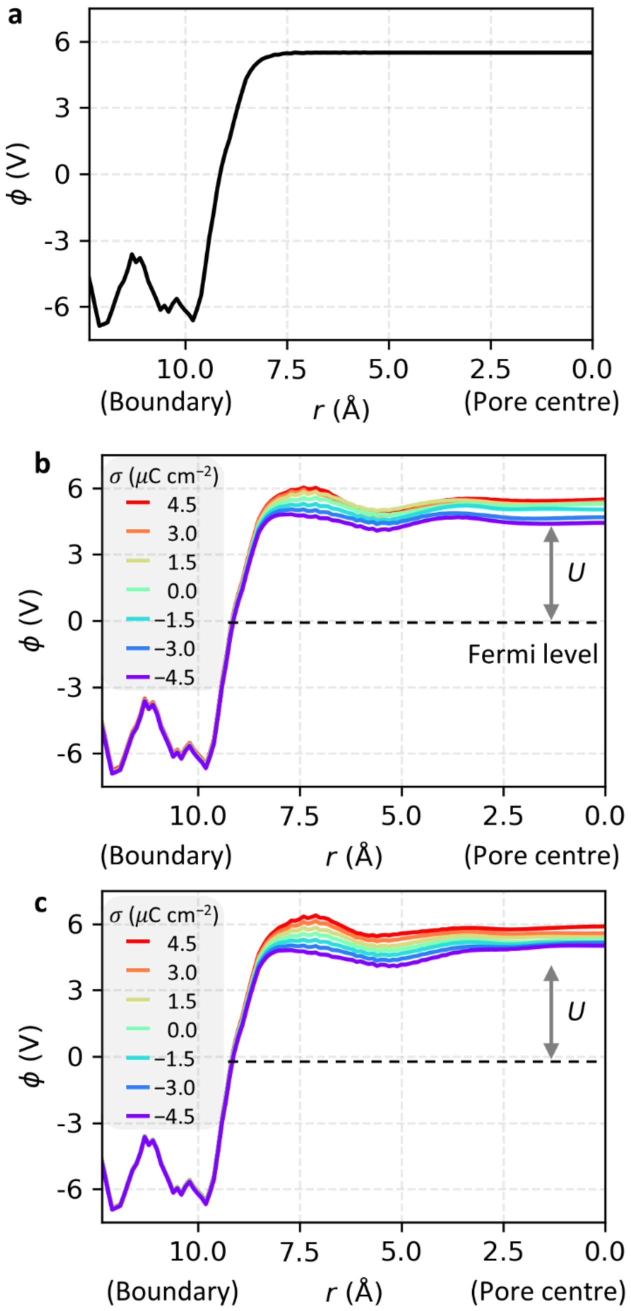

Supplementary Fig. 2. Electrostatic potential profiles (ϕ) with respect to the radial distance (r) in the cylinder varying the surface charge density (σ). The ϕ is aligned with the Fermi level and the absolute electrode potential (U) is defined as the magnitude of the plateau potential at the pore centre region. **a**, The profiles in vacuum. **b,** The profiles following the co-ion removal mechanism. **c,** The profiles following the counterion insertion mechanism.

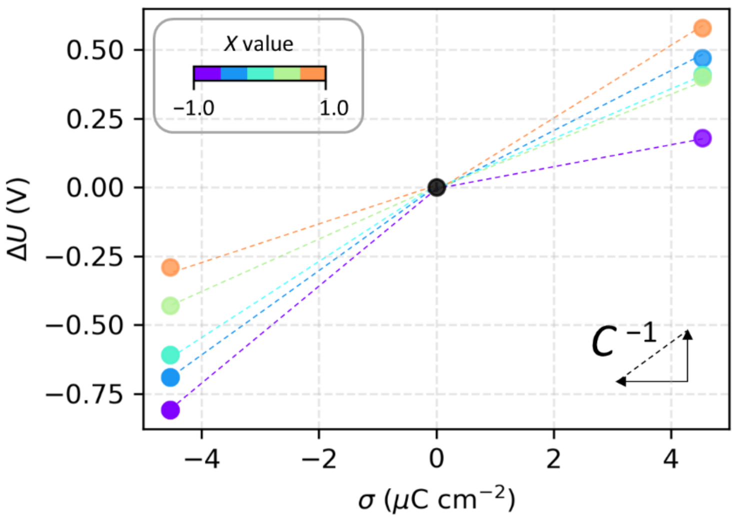

Supplementary Fig. 3. The surface charge density (σ)–biased potential (ΔU) curve of the $Cu_3(HHTP)_2$ electrochemical interface with respect to the X parameter. The differential capacitance (C) values are summarised in the main text (**Table 1**).

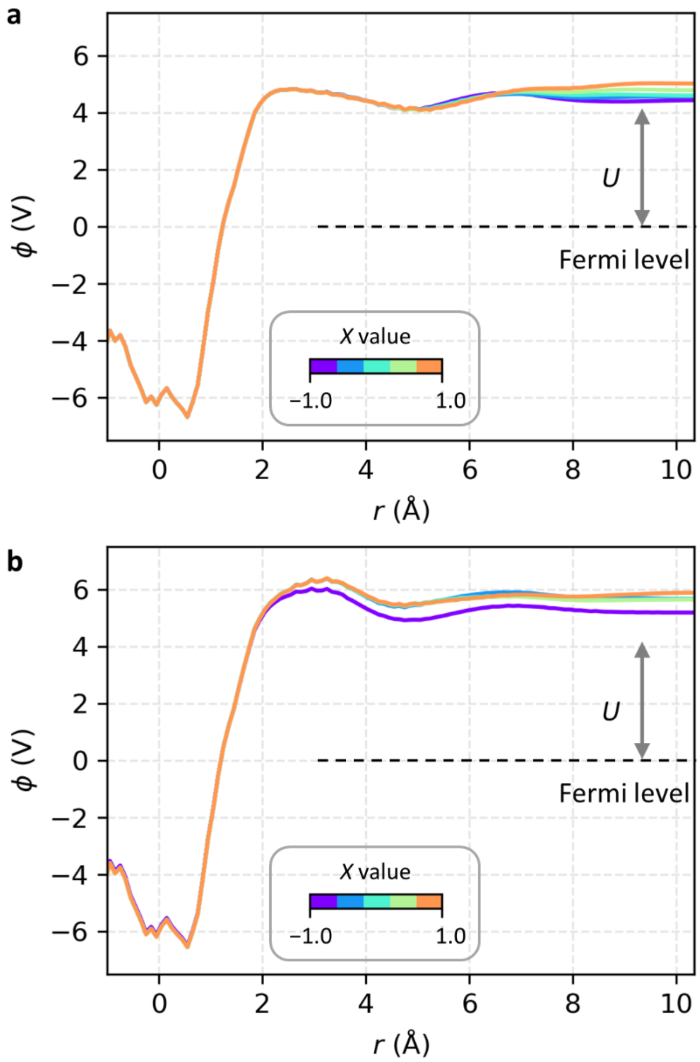

Supplementary Fig. 4. Electrostatic potential profiles (ϕ) with respect to the radial distance (r) in the cylinder varying the X value. The ϕ is aligned with the Fermi level and the absolute electrode potential (U) is defined as the magnitude of plateau potential at the pore centre region. **a**, The profiles when surface charge density (σ) is −4.5 μC cm^{-2}. **b,** The profiles when σ is +4.5 μC cm^{-2}.

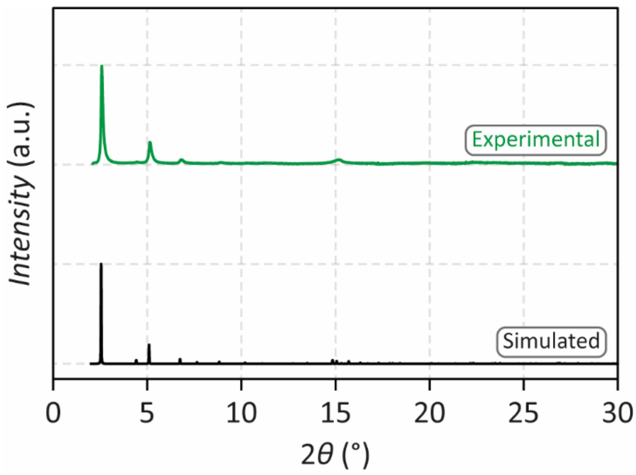

Supplementary Fig. 5. Experimental PXRD pattern from $Cu_3(HHTP)_2$ synthesised in this work (green), compared to the simulated PXRD pattern for $Cu_3(HHTP)_2$ with a hexagonal eclipsed (*P*6/*mmm*) crystal structure (black).

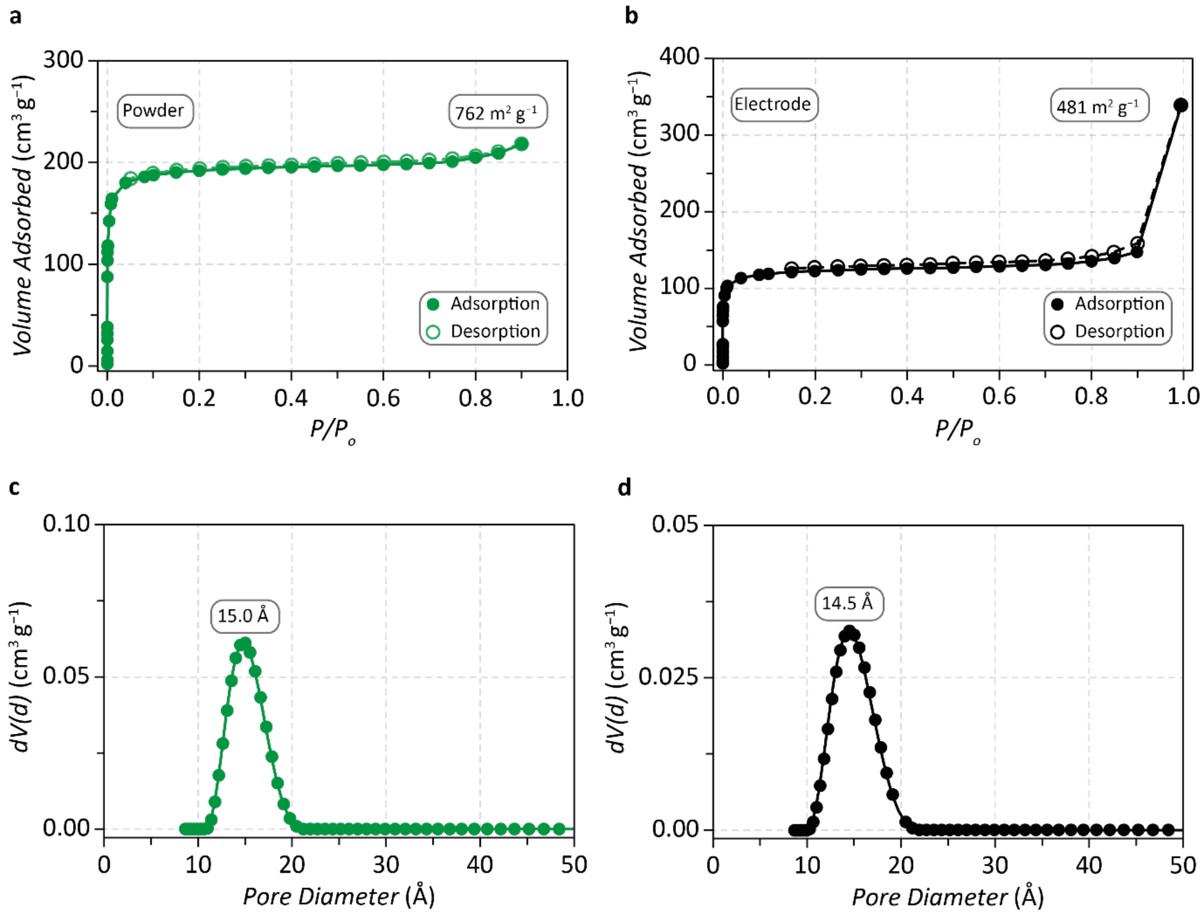

Supplementary Fig. 6. a, 77 K N_2 sorption isotherm from $Cu_3(HHTP)_2$ powder (green) synthesised in this work. From this isotherm, an experimental BET area of 762 m^2 g^{-1} was determined. **b,** 77 K N_2 sorption isotherm from $Cu_3(HHTP)_2$ electrodes (black) that were used in this work for electrochemical measurements. From this isotherm, an experimental BET area of 481 m^2 g^{-1} was determined. The approximate BET area of $Cu_3(HHTP)_2$ in the electrodes was then obtained by removing the contributions from acetylene black (62 m^2 g^{-1}) and PTFE (assumed negligible surface area). Based on a final film composition of 85 wt% $Cu_3(HHTP)_2$, 10 wt% acetylene black, and 5 wt% PTFE, an approximate BET area of 551 m^2 g^{-1} was obtained for $Cu_3(HHTP)_2$ in the electrodes. This value is 27% lower than for the powder and indicates that PTFE and/or acetylene black is blocking some of the MOF porosity and reducing its available surface area. This BET area was used when calculating experimental areal capacitance values. **c,** Experimental pore size distribution of $Cu_3(HHTP)_2$ powder determined by QSDFT modelling of the N_2 sorption isotherm with a cylindrical pore carbon model. This shows that $Cu_3(HHTP)_2$ has an experimental pore size of approximately 15.0 Å. **d,** Experimental pore size distribution of $Cu_3(HHTP)_2$ electrodes determined by QSDFT modelling of the N_2 sorption isotherm with a cylindrical pore carbon model. This shows that $Cu_3(HHTP)_2$ electrodes have an experimental pore size of approximately 15.0 Å.

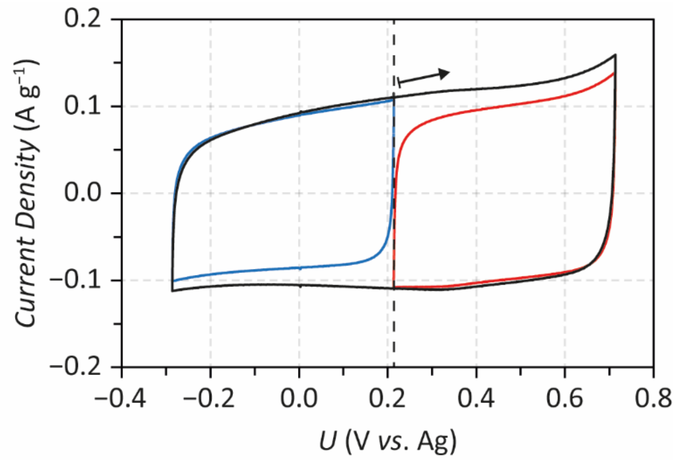

Supplementary Fig. 7. Cyclic voltammetry (CV) data obtained at a scan rate of 1 mV s^{-1} from a three-electrode cell (Cell 1) assembled with a $Cu_3(HHTP)_2$ composite working electrode, a YP80F oversized counter electrode, a Ag *pseudo*-reference electrode, and 1 M NEt_4BF_4 in acetonitrile electrolyte. The open circuit voltage (OCV) is indicated by the dashed black line. Data was acquired by scanning to +0.5 V *vs*. OCV (red) −0.5 V *vs*. OCV (blue) and across the full potential window (black). The direction of scanning is indicated by the arrow.

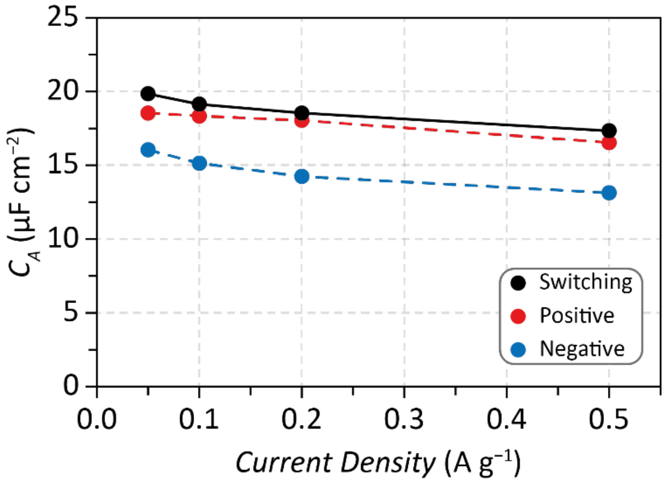

Supplementary Fig. 8. Rate capability data calculated from galvanostatic charge-discharge profiles obtained from a three-electrode cell (Cell 1), assembled with a $Cu_3(HHTP)_2$ composite working electrode, a YP80F oversized counter electrode, a Ag *pseudo*-reference electrode, and 1 M NEt_4BF_4 in acetonitrile electrolyte. All areal capacitance values were calculated from galvanostatic charge-discharge curves. This shows how the areal capacitance (C_A) varies with current density (A g^{-1}) upon charging to +0.5 V *vs*. OCV (red) −0.5 V *vs*. OCV (blue) and across the full potential window (from +0.5 V to −0.5 V; black and denoted as "Switching"). This reveals the kinetic behaviour of the system. The capacitance values are summarised in the **Supplementary Table 1**.

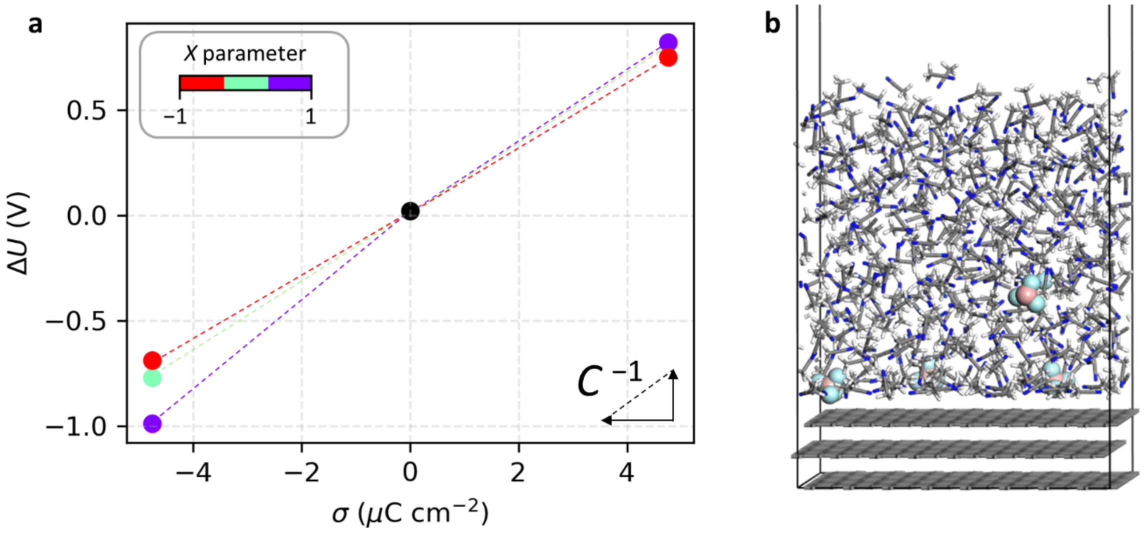

Supplementary Fig. 9. Electrochemical interface of graphite. **a,** The surface charge density (σ)–biased potential (ΔU) curve with respect to the ion-exchange ratio on the graphite electrode. The slope indicates the inverse differential capacitance (C), and it is calculated to be 5–7 μF cm^{-2}. **b,** A representative model to study the electrochemical interface of graphite.

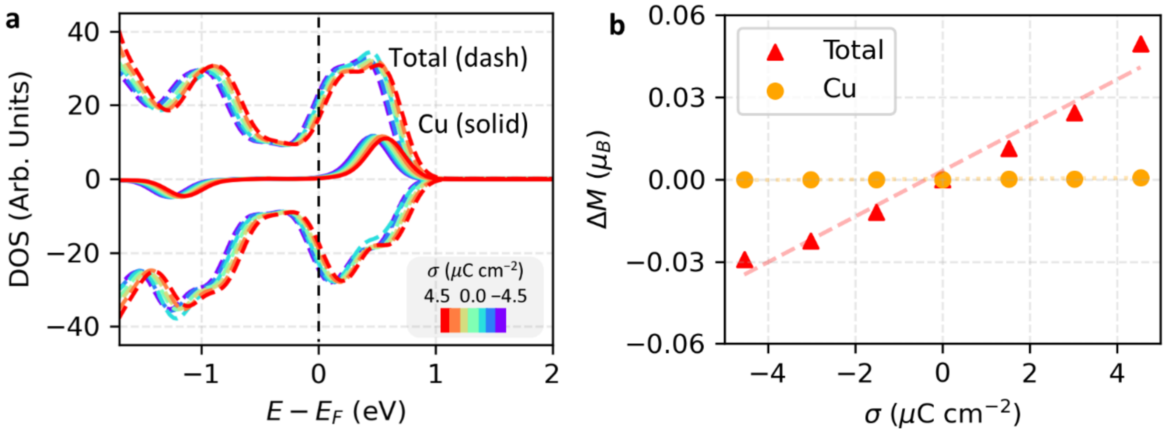

Supplementary Fig. 10. a, The density of states (DOS) near the Fermi level is shown at each surface charge density (σ). The total DOS is plotted with the dashed line, while the partial DOS of Cu is plotted with the solid line. The simulation cell has an anti-ferromagnetic spin configuration that only one configuration is shown for the Cu atom. **b,** Difference of magnetic moment (ΔM) referring to the point of zero charge (PZC) with respect to the σ. The total ΔM is per simulation cell. All figures are constructed following the co-ion removal mechanism.

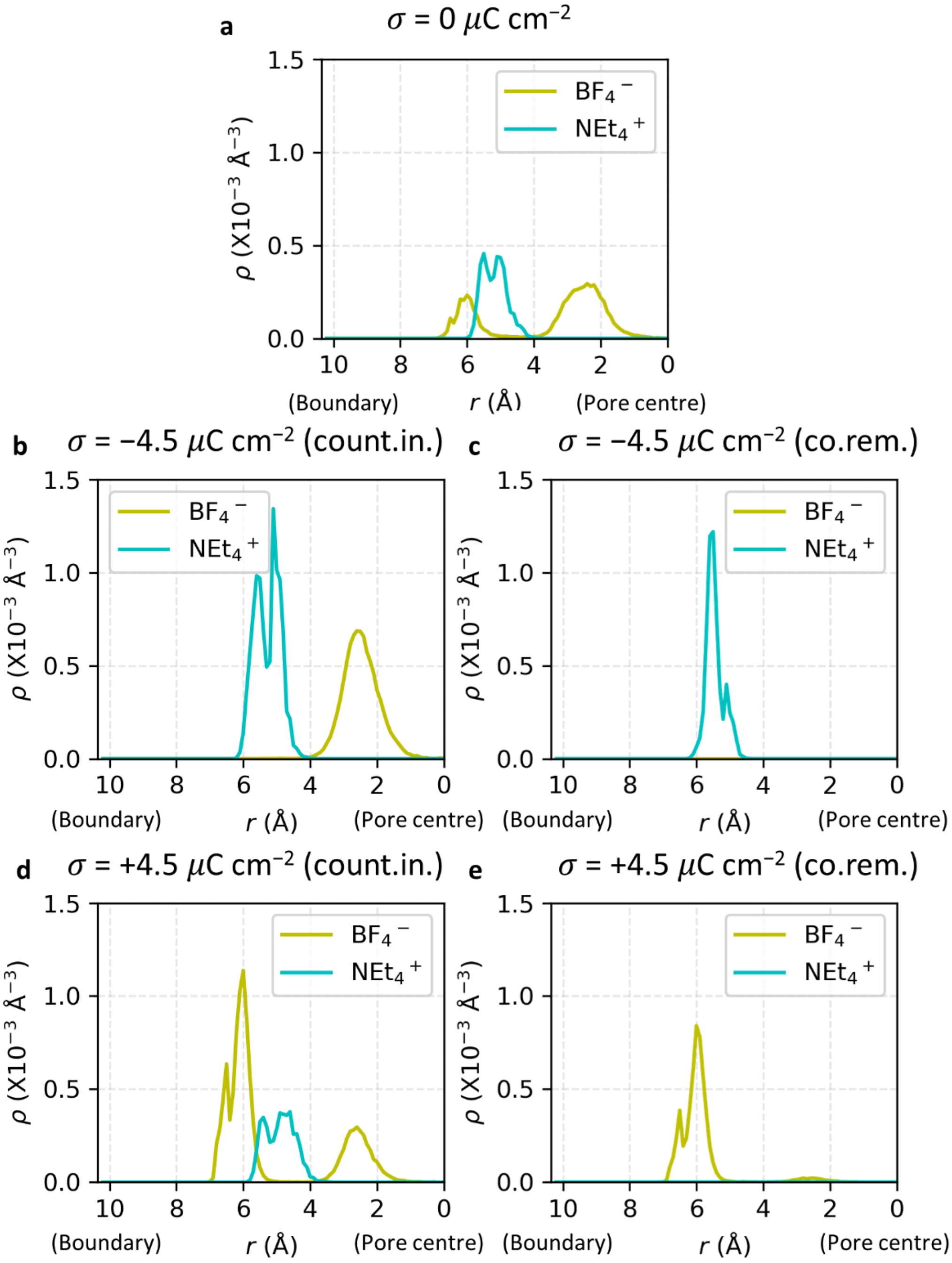

Supplementary Fig. 11. a–e, Density (ρ) of cations and anions in $Cu_3(HHTP)_2$ are shown with respect to the radial distance (r) from the centre of the MOFs. The density profiles are shown at surface charge density (σ) is 0 μC cm^{-2} (**a**), σ is −4.5 μC cm^{-2} following the counterion insertion mechanism (count.in.) (**b**), or the co-ion removal mechanism (co.rem.) (**c**), σ is +4.5 μC cm^{-2} following the count.in. mechanism (**d**), or the co.rem. mechanism (**e**).

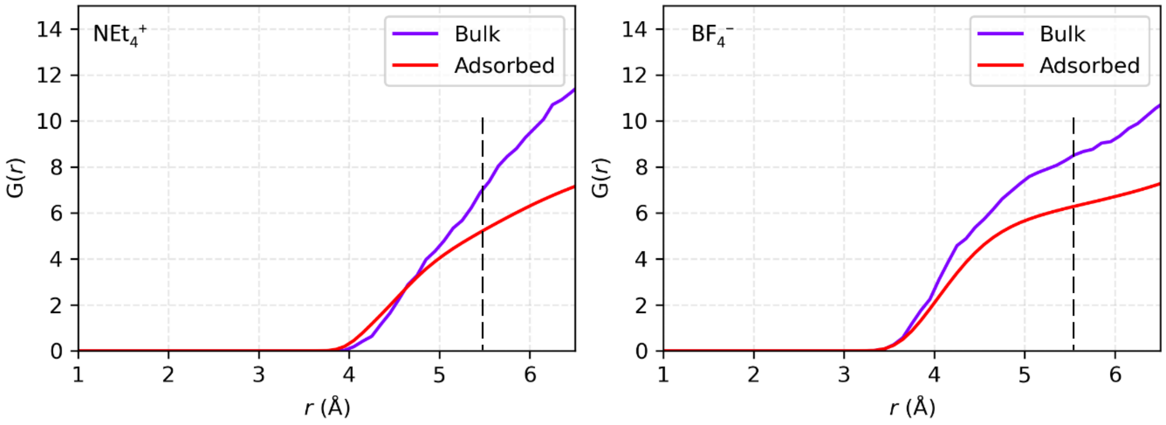

Supplementary Fig. 12. The integrated radial distribution function (RDF) between the ions and acetonitrile molecules in the bulk electrolyte or in the cylindrical pores at the point of zero charge (PZC). The radial distance (r) is calculated between B atom of BF_4^- and C atom of hydrocarbon fragment in acetonitrile. The r between the NEt_4^+ and the acetonitrile is calculated between the two N atoms. The coordination number is read using $\mathrm{G}(r) = \int 4\pi r^2 \mathrm{g}(r) dr$ at 5.5 Å.

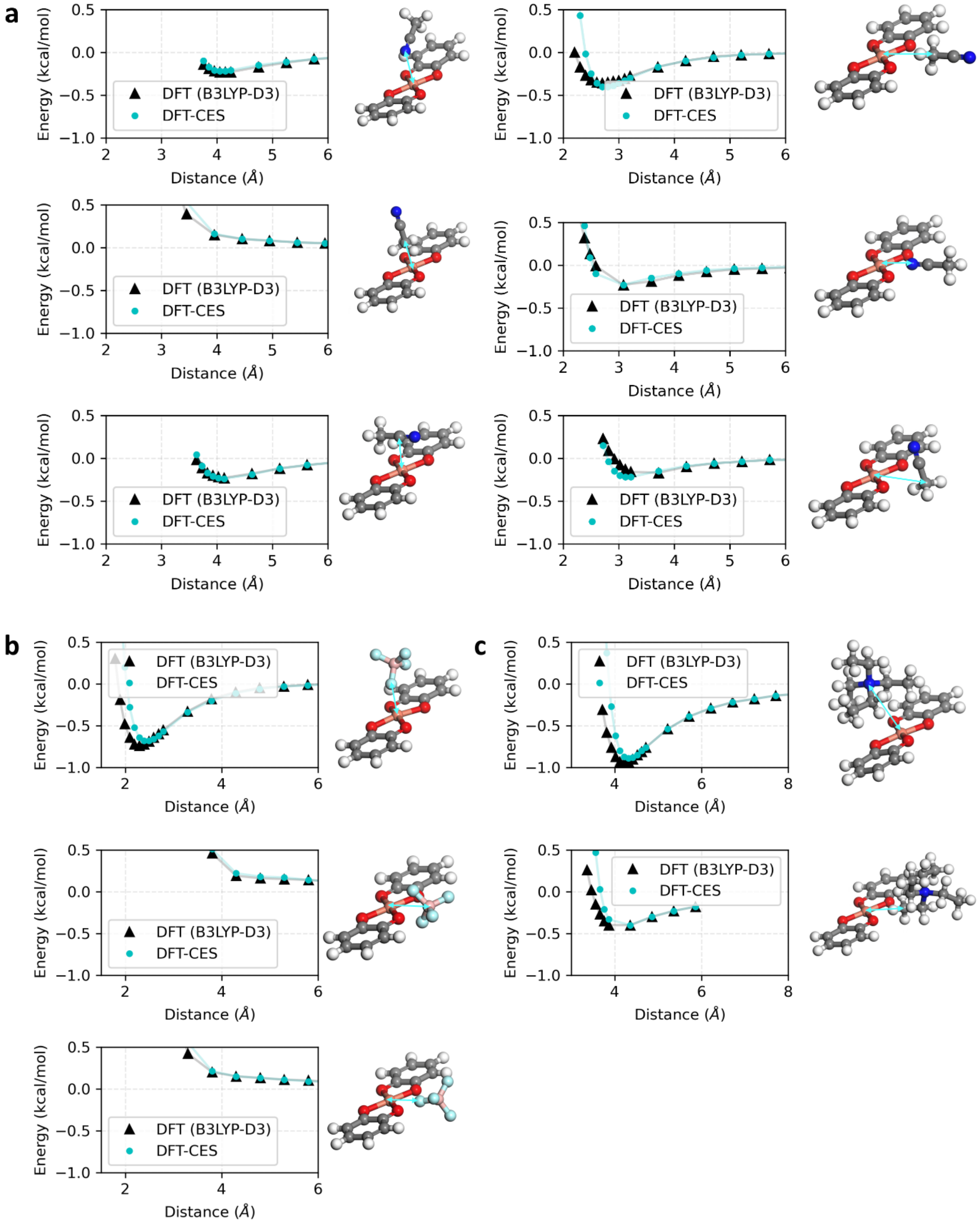

Supplementary Fig. 13. The binding energy from the DFT-CES method, which benchmark the DFT binding energy. $Cu_3(HHTP)_2$ is represented as one Cu atom, four O atoms, and 2 benzene molecules. **a–c,** The binding energy between the MOF fragment and acetonitrile (**a**), BF_4^- (**b**), and NEt_4^+ (**c**) with multiple geometries. The Lennard-Jones potential parameter of which functional is $V(r) = \varepsilon\left[\left(\frac{R}{r}\right)^{12} - \left(\frac{R}{r}\right)^{6}\right]$ for the Cu atom is set as ε for 0.867 eV and R for 2.36 Å.

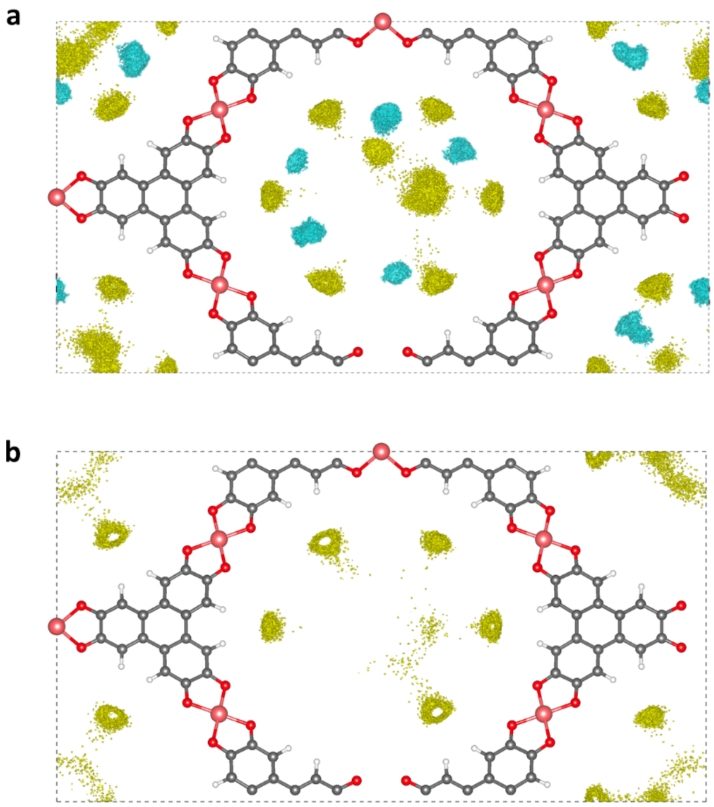

Supplementary Fig. 14. a–b, Isosurface of the time-averaged ion distribution is shown when surface charge density (σ) is +4.5 μC cm^{-2} following the counterion insertion mechanism (**a**) and co-ion removal mechanism (**b**). The distribution of cations or anions is coloured cyan or yellow using their centre of mass, respectively. The isosurface level is 0.001 e $bohr^{-3}$. The acetonitrile distribution is omitted to provide a clear view.

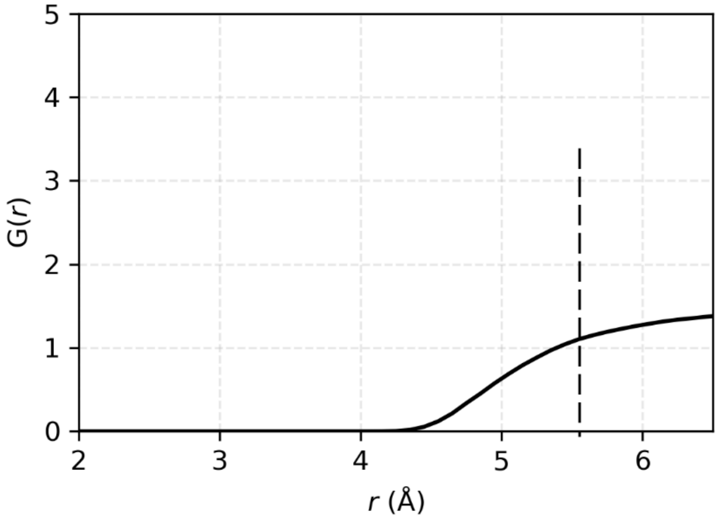

Supplementary Fig. 15. The integrated radial distribution function between BF_4^- at the pore centre region and NEt_4^+. The radial distance (r) is calculated between B atom of BF_4^- and N atom of the NEt_4^+. The coordination number is read using $\mathrm{G}(r) = \int 4\pi r^2 \mathrm{g}(r) dr$ at 5.5 Å.

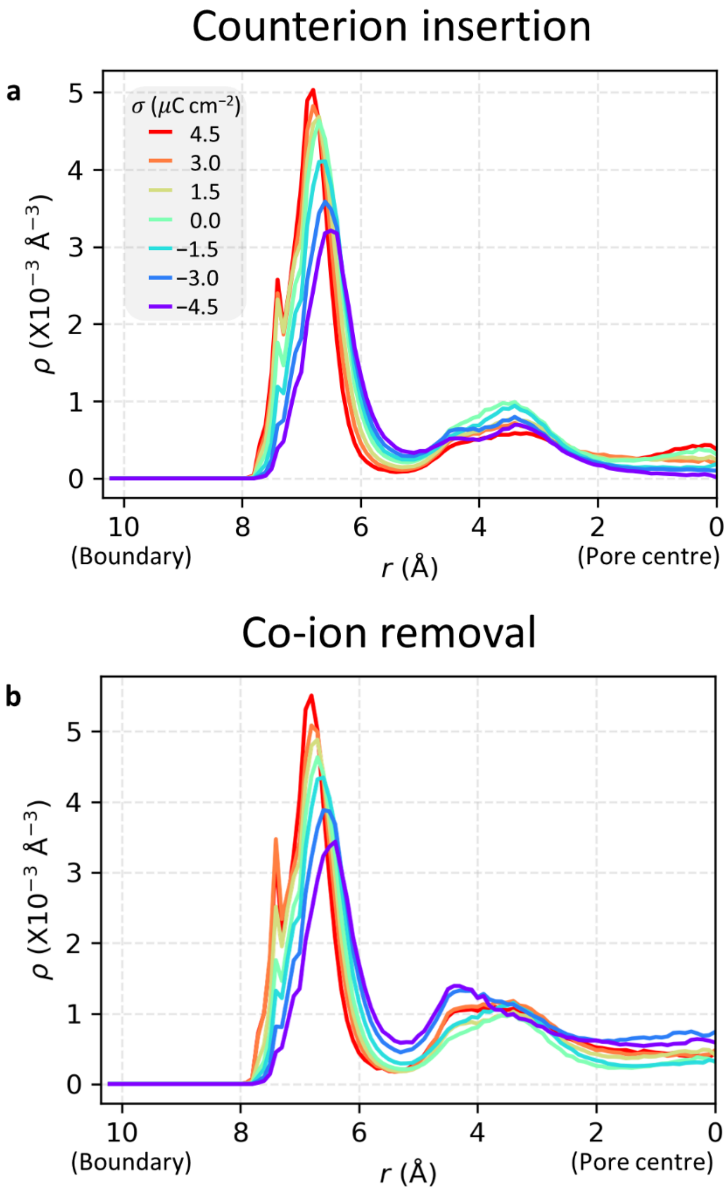

Supplementary Fig. 16. Solvent density (ρ) profile with respect to the radial distance (r) in the cylinder, varying the surface charge density (σ). The N atom of acetonitrile is averaged to calculate the ρ. Thus, the rotating behaviour along the excess charges on the MOFs is figured out here, i.e. the N atom is repelled from the surface by charging the surface from σ is +4.5 μC cm^{-2} to σ is −4.5 μC cm^{-2} because of the electrostatic repulsion between the electrode and N atom. **a,** The counterion insertion mechanism is considered. **b,** The co-ion removal mechanism is considered.

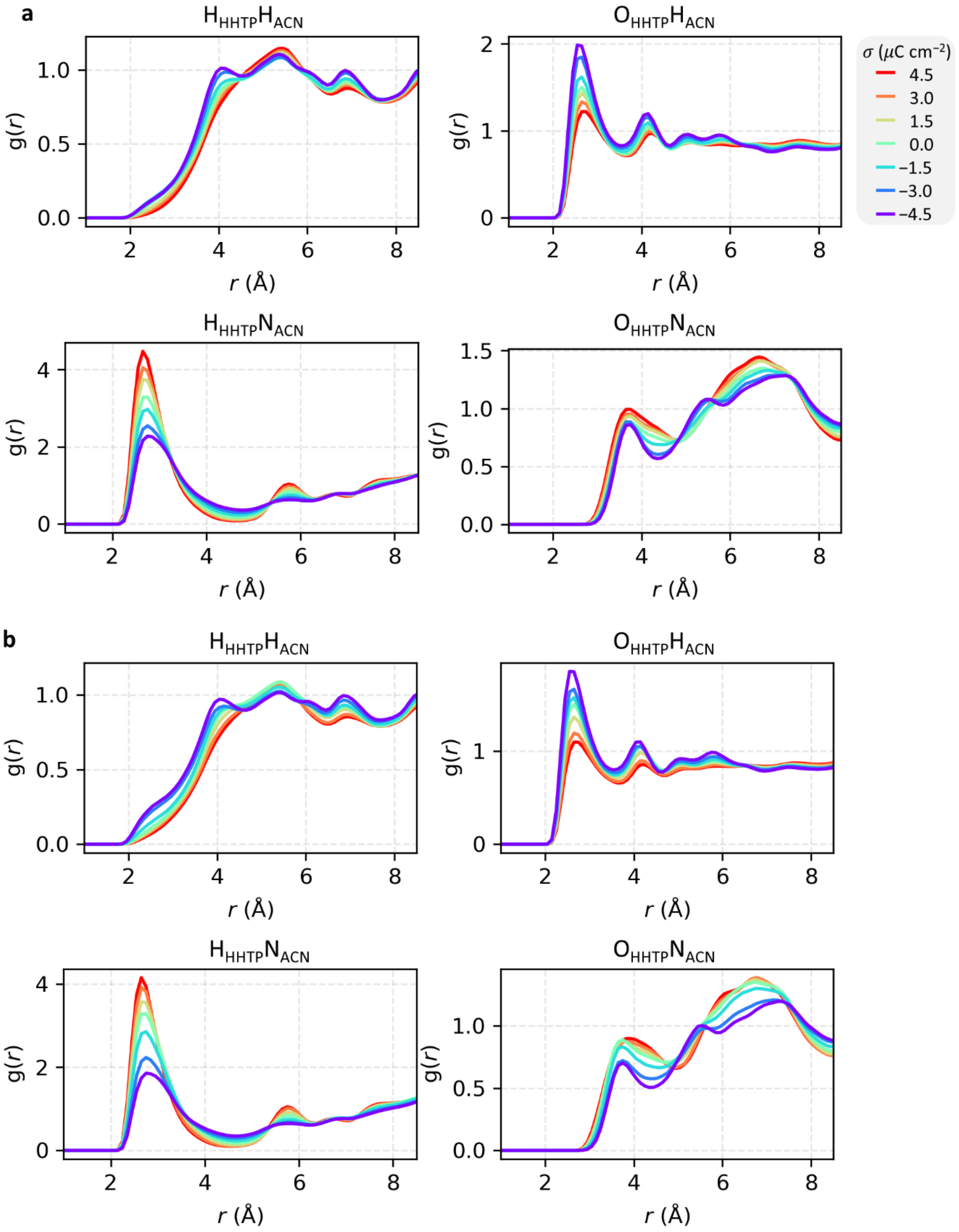

Supplementary Fig. 17. Radial distribution function, g(r), with respect to the radial distance (r) between the H or O atom of $Cu_3(HHTP)_2$ (HHTP) and H or N atom of the acetonitrile (ACN) molecules. **a,** The counterion insertion mechanism is considered. **b,** The co-ion removal mechanism is considered.

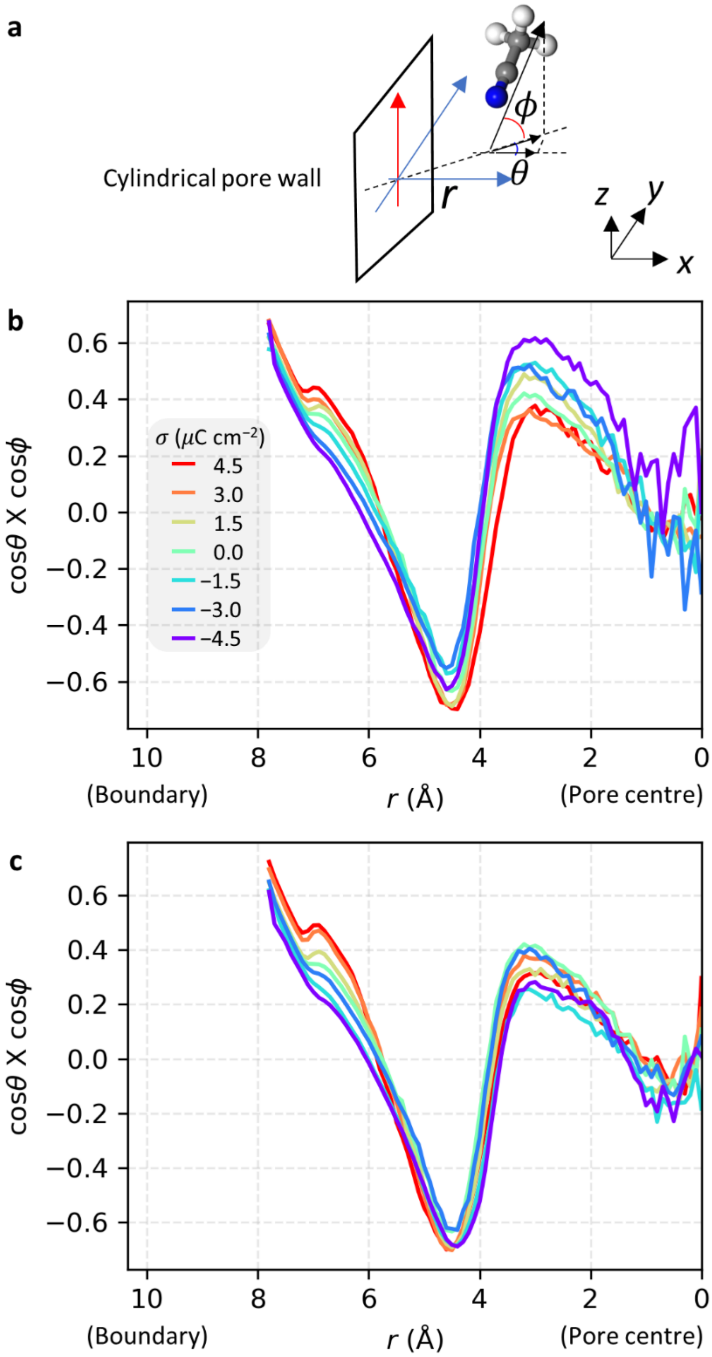

Supplementary Fig. 18. The molecular orientation of the acetonitrile. **a,** A schematic figure to define the molecular orientation where the molecular vector is defined along the N-C bond. Two angles are defined where the ϕ is an angle between the acetonitrile and the xy plane. The θ is the angle between the projected vector on the xy plane and the surface normal vector of the cylindrical pore wall. **b–c,** The $\cos\phi \times \cos\theta$ is used as the order parameter and it is plotted with respect to the radial distance (r) in the cylinder following the counterion insertion mechanism (**b**) or the co-ion removal mechanism (**c**).

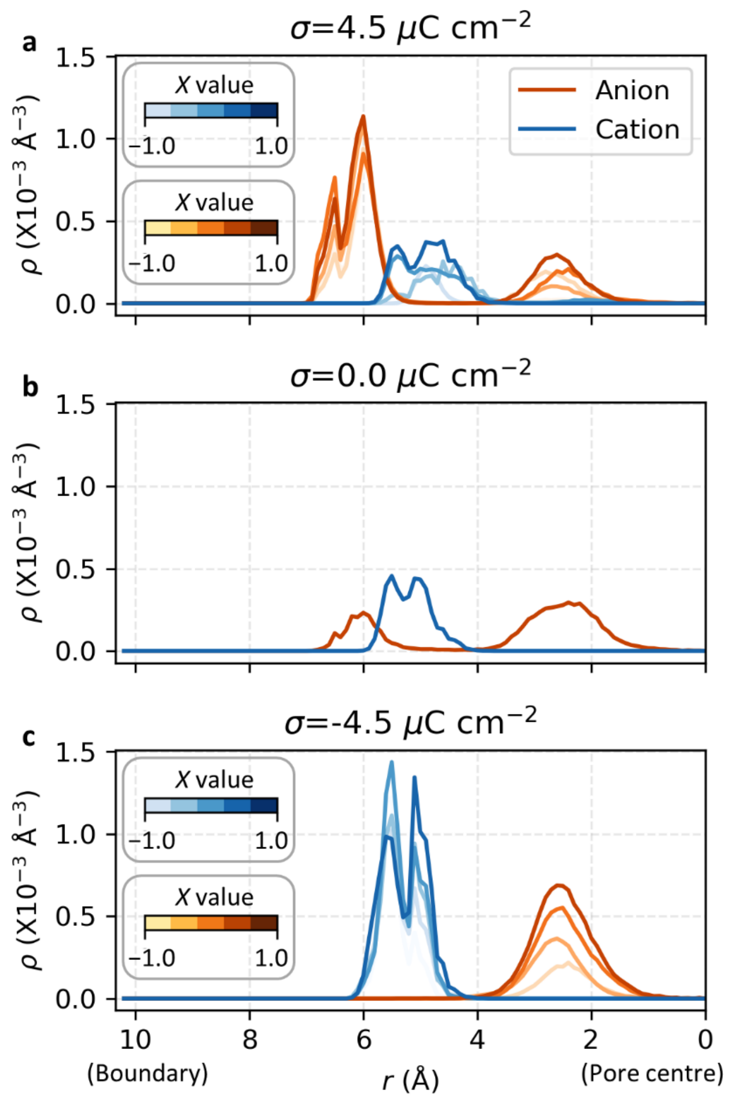

Supplementary Fig. 19. **a–c,** Density (ρ) of cations and anions in $Cu_3(HHTP)_2$ are shown with respect to the radial distance (r) from the centre of the MOFs using their centre of mass. The saturation of colour indicates the density of components at each X value. It is plotted when surface charge density (σ) is +4.5 μC cm^{-2} (**a**), σ is 0.0 μC cm^{-2} (**b**), and σ is −4.5 μC cm^{-2} (**c**).

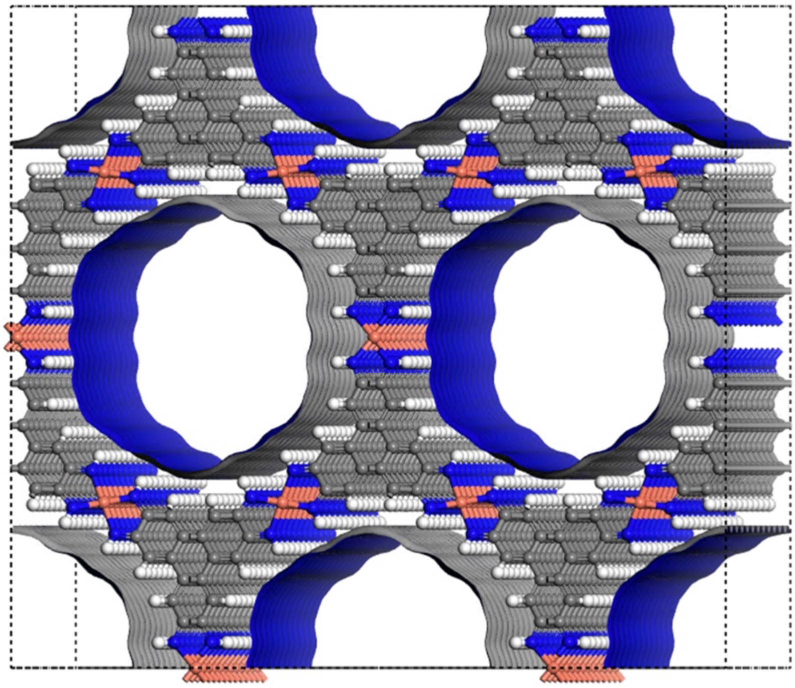

Supplementary Fig. 20. Surface area of the $Cu_3(HITP)_2$ MD simulation cell. The surface area is estimated as 1333 m^2 g^{-1} by the Connolly surface area employing a probe molecule with a kinetic diameter of 3.68 Å, equivalent to the kinetic diameter of N_2.

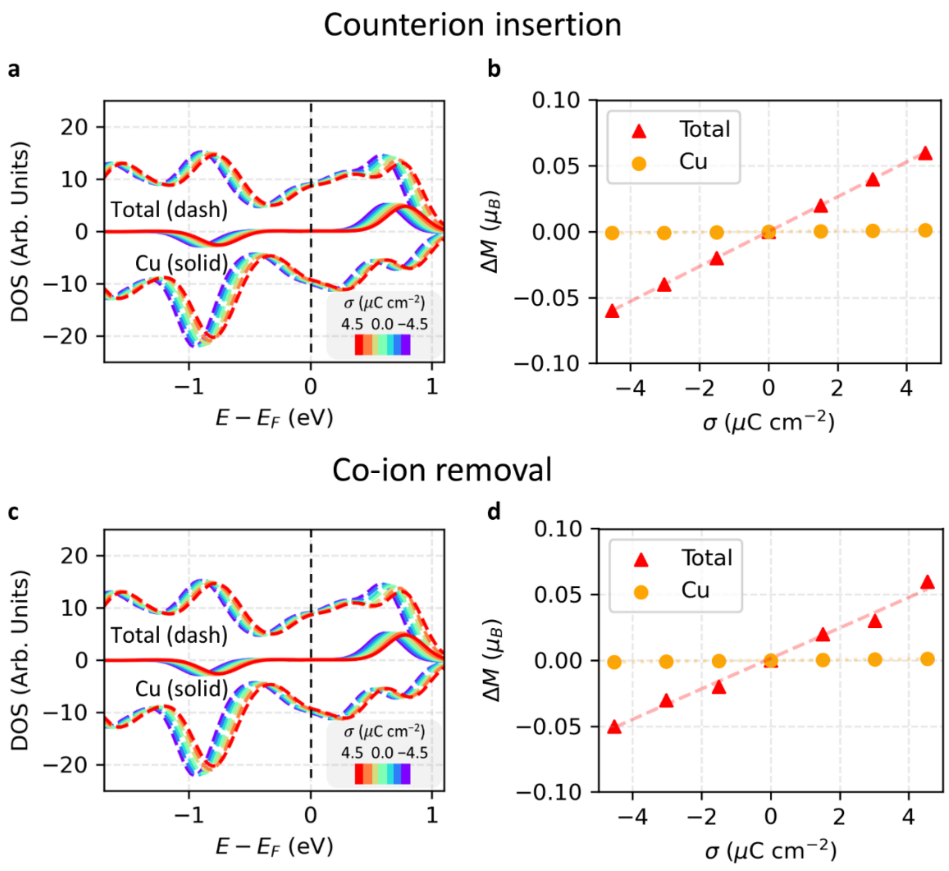

Supplementary Fig. 21. a, Density of states (DOS) of $Cu_3(HITP)_2$ at the electrochemical interface near the Fermi level is shown at each surface charge density (σ) following the counterion insertion mechanism. The total DOS is plotted with the dashed line, while the partial DOS of Cu is plotted with the solid line. The simulation cell has an anti-ferromagnetic spin configuration that only one configuration is shown for the Cu atom. **b,** Difference of magnetic moment (ΔM) referring to the PZC with respect to the σ following the counterion insertion mechanism for $Cu_3(HITP)_2$ at the electrochemical interface. The total ΔM is per simulation cell. The M of Cu is 0.6 μ_B. **c–d,** The same plots are shown following the co-ion removal mechanism.

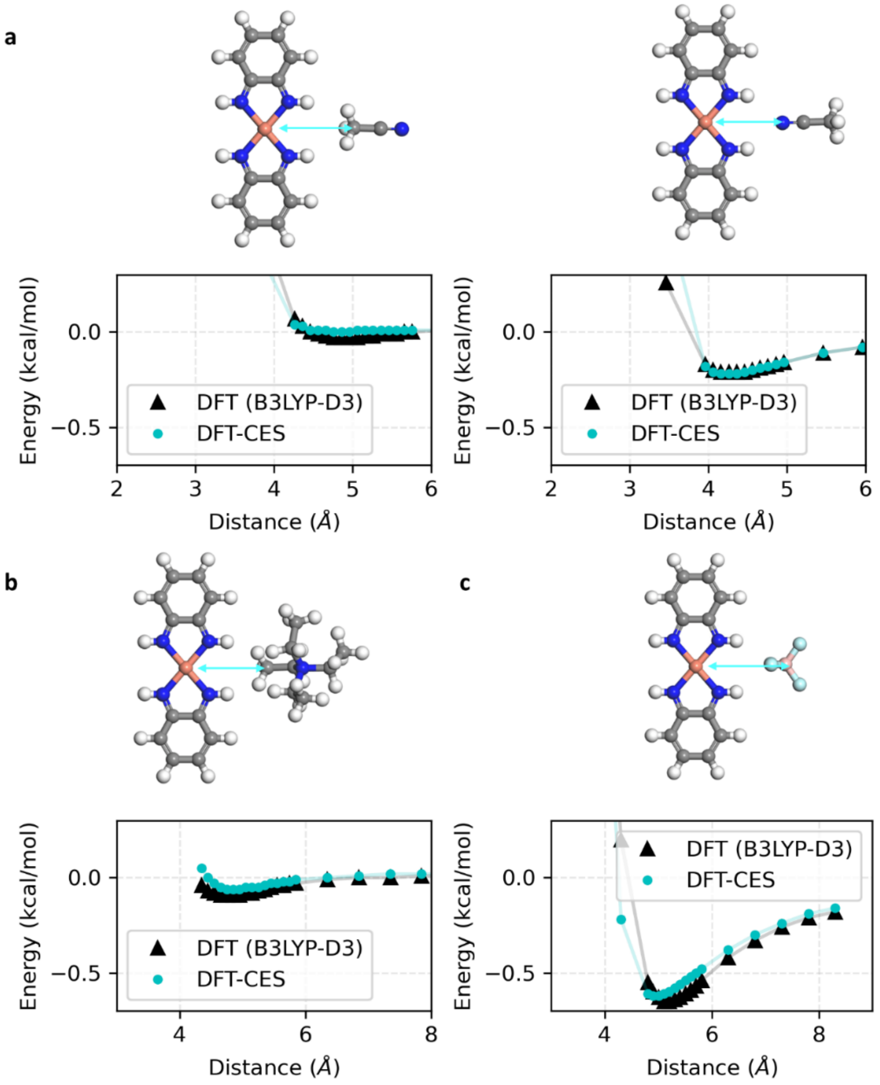

Supplementary Fig. 22. The binding energy from the DFT-CES method, which benchmark the DFT binding energy. $Cu_3(HITP)_2$ is represented as one Cu atom, four N atoms, four H atoms, and 2 benzene molecules. **a**–**c**, The binding energy between the MOF fragment and acetonitrile (**a**), BF_4^- (**b**), and NEt_4^+ (**c**) with multiple geometries. The Lennard-Jones potential parameter of which functional is $V(r) = \varepsilon\left[\left(\frac{R}{r}\right)^{12} - \left(\frac{R}{r}\right)^{6}\right]$ for the Cu atom is set as ε for 0.01 eV and R for 7.1 Å.

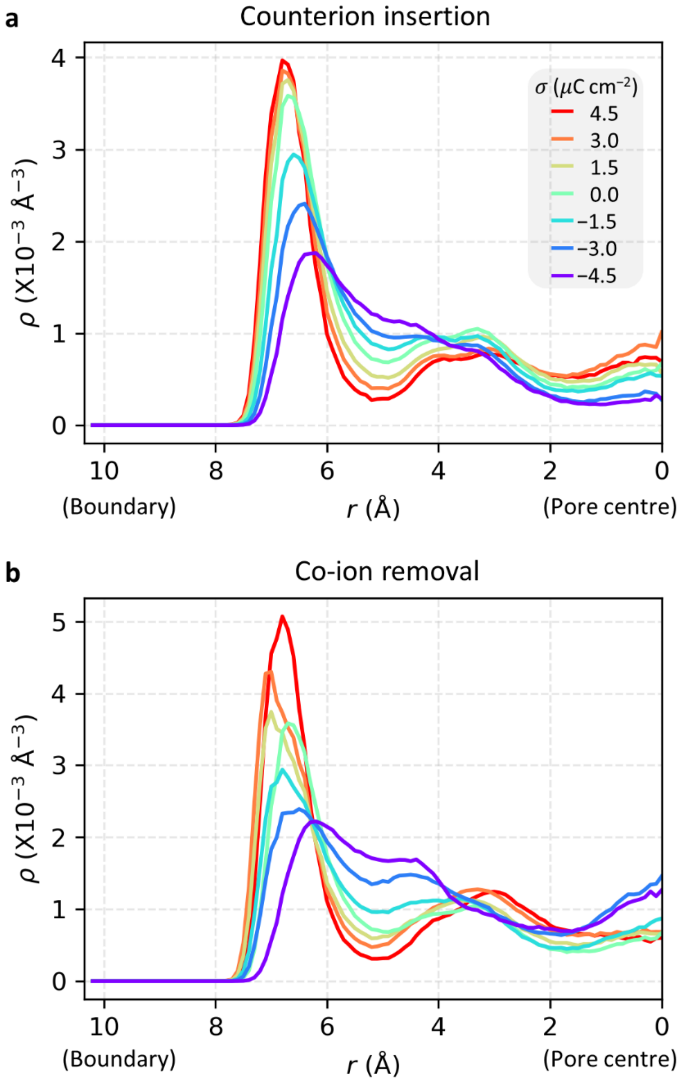

Supplementary Fig. 23. Solvent density (ρ) profile at the $Cu_3(HITP)_2$ electrochemical interface with respect to the radial distance (r) in the cylinder varying the surface charge density (σ). The N atom of acetonitrile is averaged to calculate the ρ. Thus, the rotating behaviour along the excess charges on the MOFs is figured out here, i.e. the N atom is repelled from the surface by charging the surface from σ is +4.5 μC cm^{-2} to σ is −4.5 μC cm^{-2} because of the electrostatic repulsion between electrode and N atom. **a,** The counterion insertion mechanism is considered. **b,** The co-ion removal mechanism is considered.

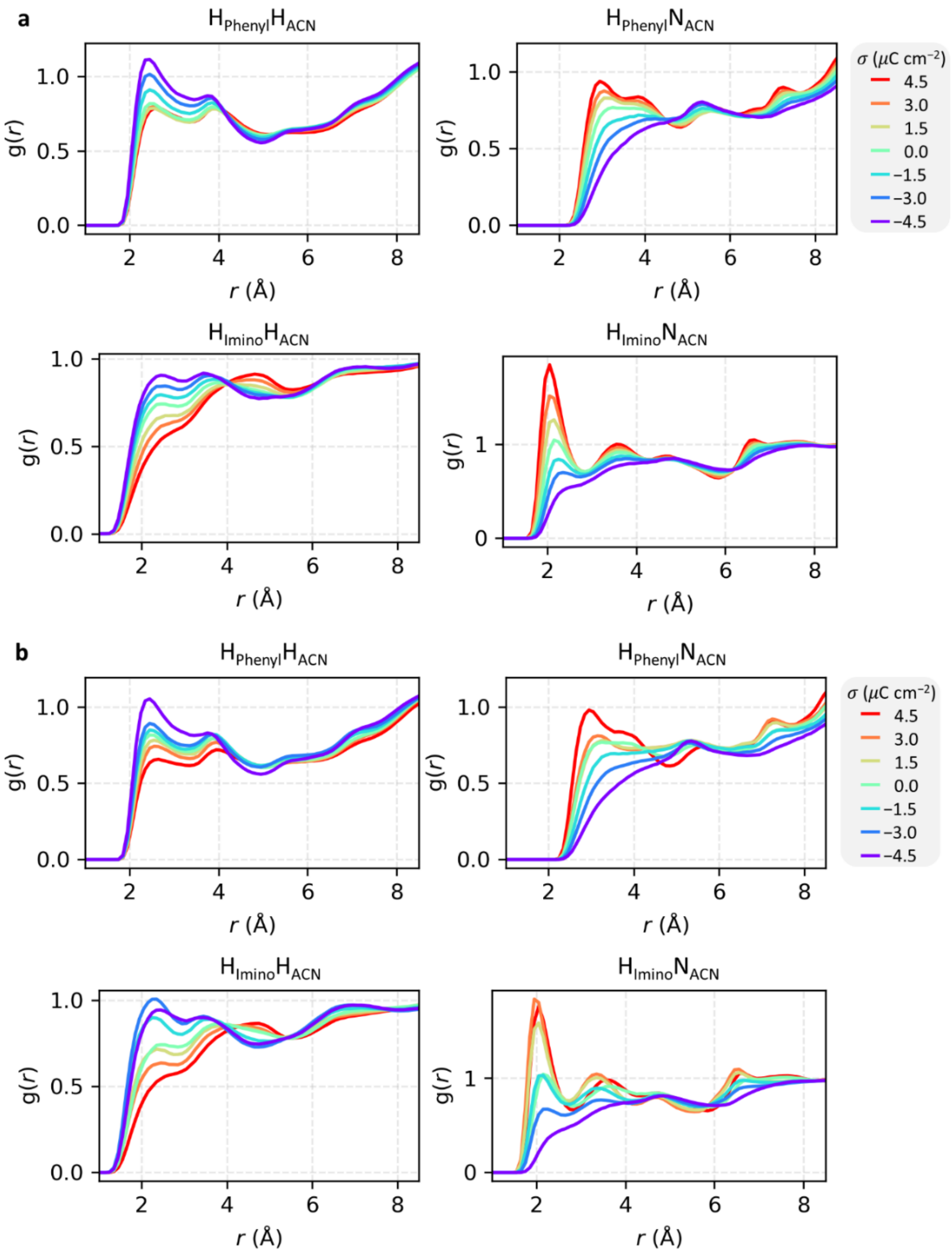

Supplementary Fig. 24. Radial distribution function, g(r), with respect to the radial distance (r) between the H atom of the phenyl group in $Cu_3(HITP)_2$ (Phenyl) or H atom of the imino group in $Cu_3(HITP)_2$ (Imino) and H or N atom of the acetonitrile (ACN) molecules. **a,** The counterion insertion mechanism is considered. **b,** The co-ion removal mechanism is considered.

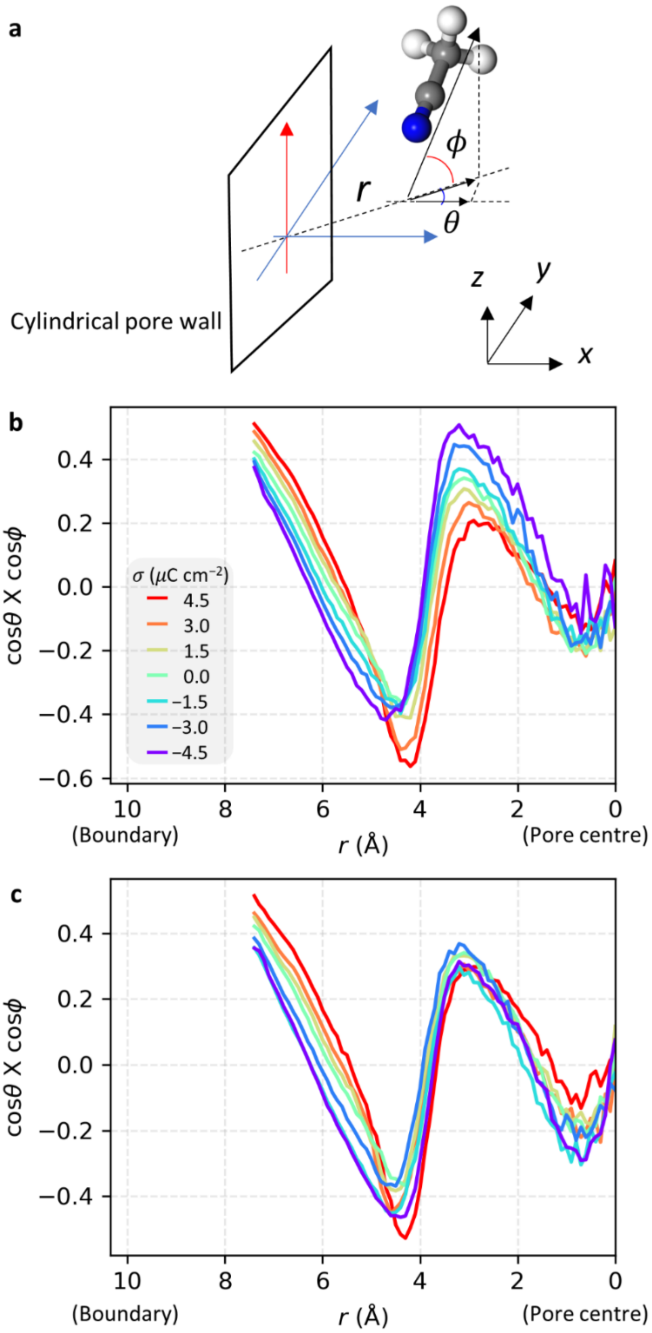

Supplementary Fig. 25. The molecular orientation of acetonitrile at the $Cu_3(HITP)_2$ electrochemical interface. **a,** A schematic figure to define the molecular orientation where the molecular vector is defined along the N-C bond. Two angles are defined where the ϕ is an angle between the acetonitrile and the xy plane. The θ is the angle between the projected vector on the xy plane and the surface normal vector of the cylindrical pore wall. **b,c,** The $\cos\phi \times \cos\theta$ is used as the order parameter and it is plotted with respect to the radial distance (r) in the cylinder following the counterion insertion mechanism (**b**) or the co-ion removal mechanism (**c**).

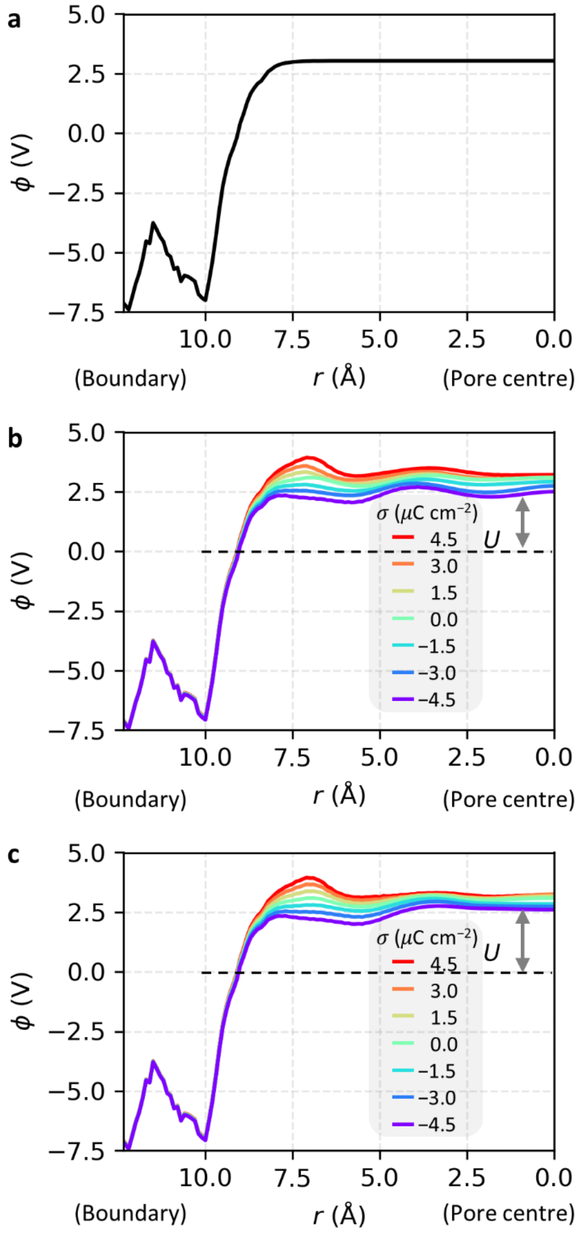

Supplementary Fig. 26. Electrostatic potential profiles (ϕ) of the $Cu_3(HITP)_2$ electrochemical interface with respect to the radial distance (r) in the cylinder varying the surface charge density (σ). The ϕ is aligned with the Fermi level and the absolute electrode potential (U) is defined as the magnitude of the plateau potential at the pore centre region. **a**, The profiles in vacuum. **b,** The profiles following the co-ion removal mechanism. **c,** The profiles following the counterion insertion mechanism.

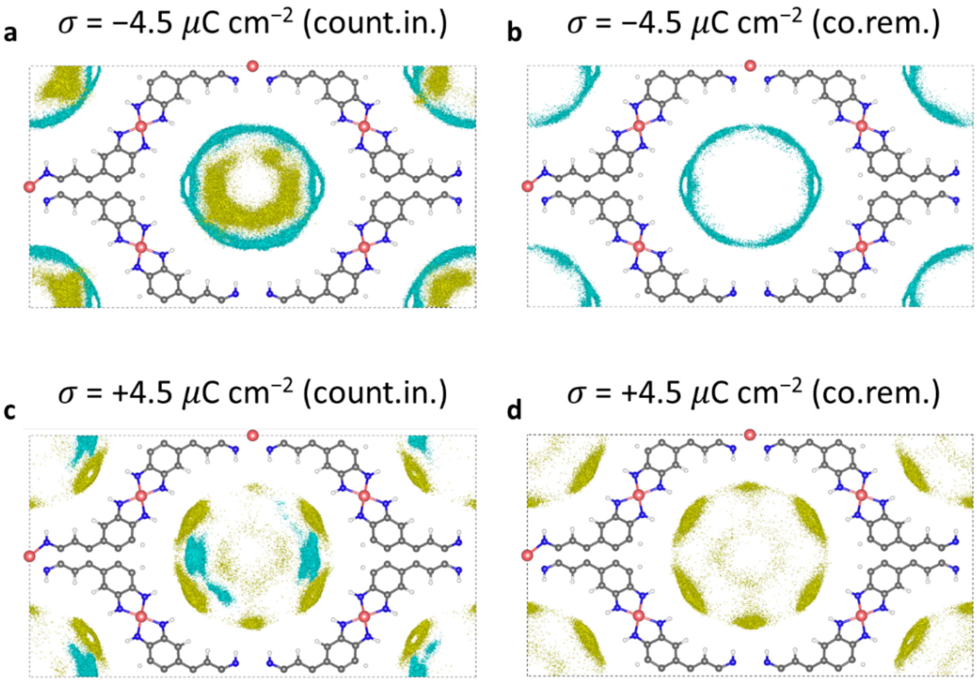

Supplementary Fig. 27. a–d, Isosurface of the time-averaged ion distribution in $Cu_3(HITP)_2$ is shown when surface charge density (σ) is −4.5 μC cm^{-2} following the counterion insertion (count.in.) mechanism (**a**) and co-ion removal (co.rem.) mechanism (**b**). It is shown when σ is +4.5 μC cm^{-2} following the count.in. mechanism (**c**) and co.rem. mechanism (**d**). The distribution of cations or anions is coloured cyan or yellow, using their centre of mass, respectively. The isosurface level is 0.001 e $bohr^{-3}$. The acetonitrile distribution is omitted to provide a clear view.

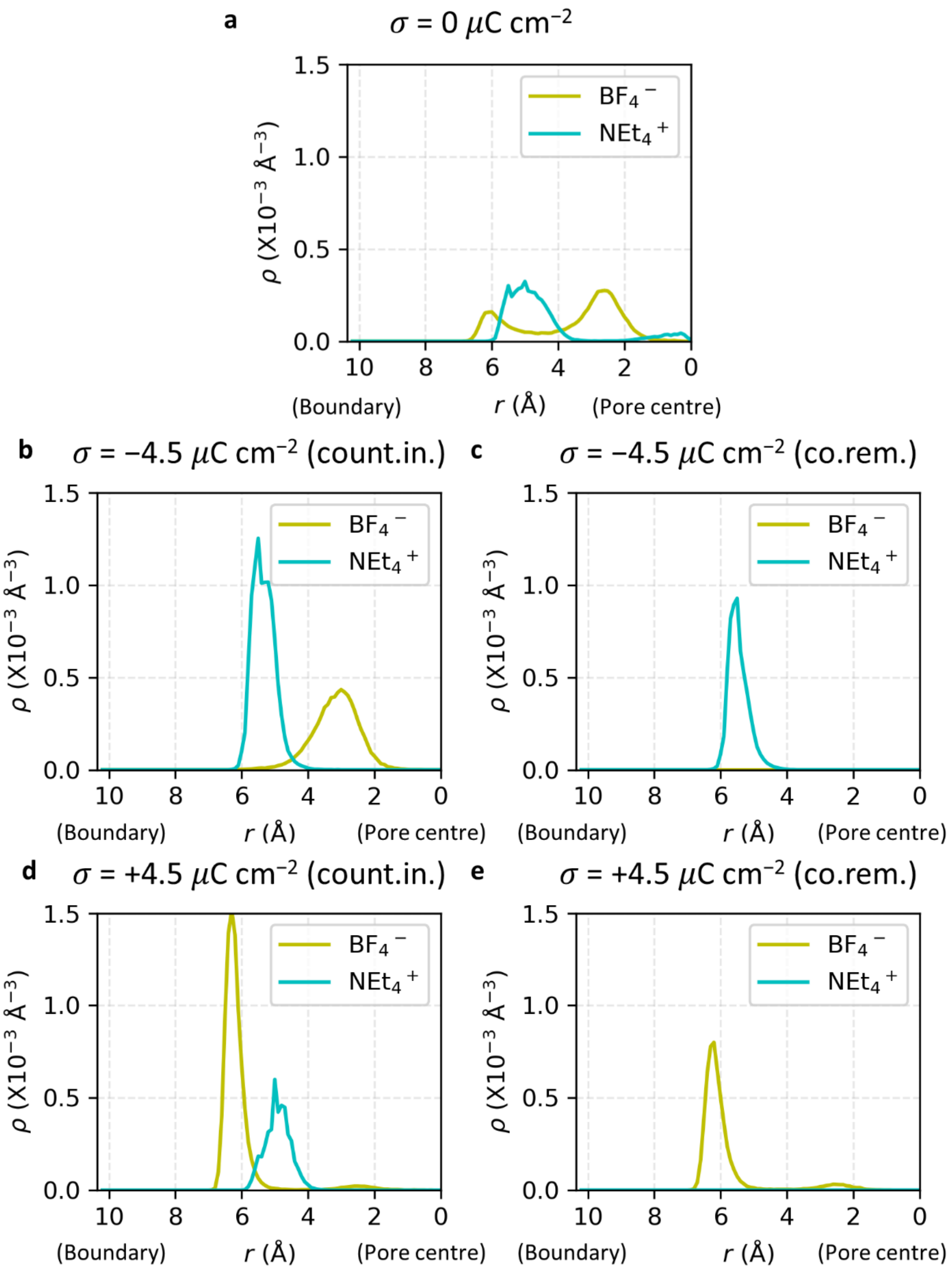

Supplementary Fig. 28. a–e, Density (ρ) of cations and anions in $Cu_3(HITP)_2$ are shown with respect to the radial distance (r) from the centre of the MOFs. The density profiles are shown at surface charge density (σ) is 0 μC cm^{-2} (**a**), σ is −4.5 μC cm^{-2} following the counterion insertion mechanism (count.in.) (**b**), or the co-ion removal mechanism (co.rem.) (**c**), σ is +4.5 μC cm^{-2} following the count.in. mechanism (**d**), or the co.rem. mechanism (**e**).

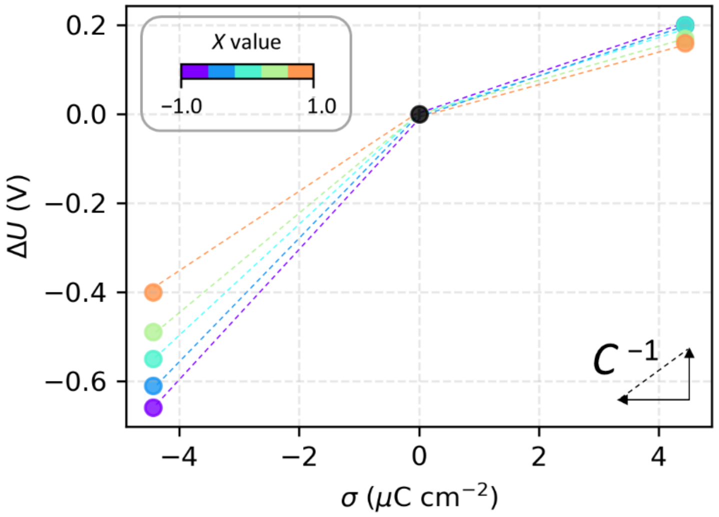

Supplementary Fig. 29. The surface charge density (*σ*)–biased potential (Δ*U*) curve of the $Cu_3(HITP)_2$ electrochemical interface with respect to the *X* parameter. The slope indicates the inverse differential capacitance (*C*). The *C* values are summarised in the main text **(Table 2)**.

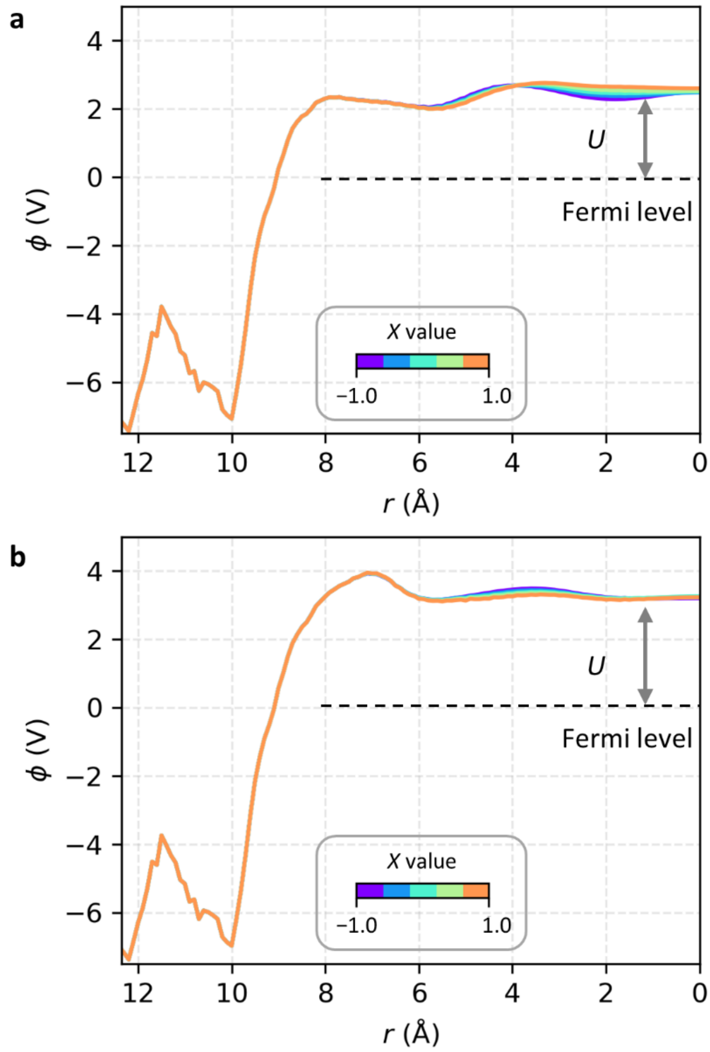

Supplementary Fig. 30. Electrostatic potential profiles (ϕ) of the $Cu_3(HITP)_2$ electrochemical interface with respect to the radial distance (r) in the cylinder varying the X value. The ϕ is aligned with the Fermi level and the absolute electrode potential (U) is defined as the magnitude of plateau potential at the pore centre region. **a**, The profiles when surface charge density (σ) is −4.5 μC cm^{-2}. **b,** The profiles when σ is +4.5 μC cm^{-2}.

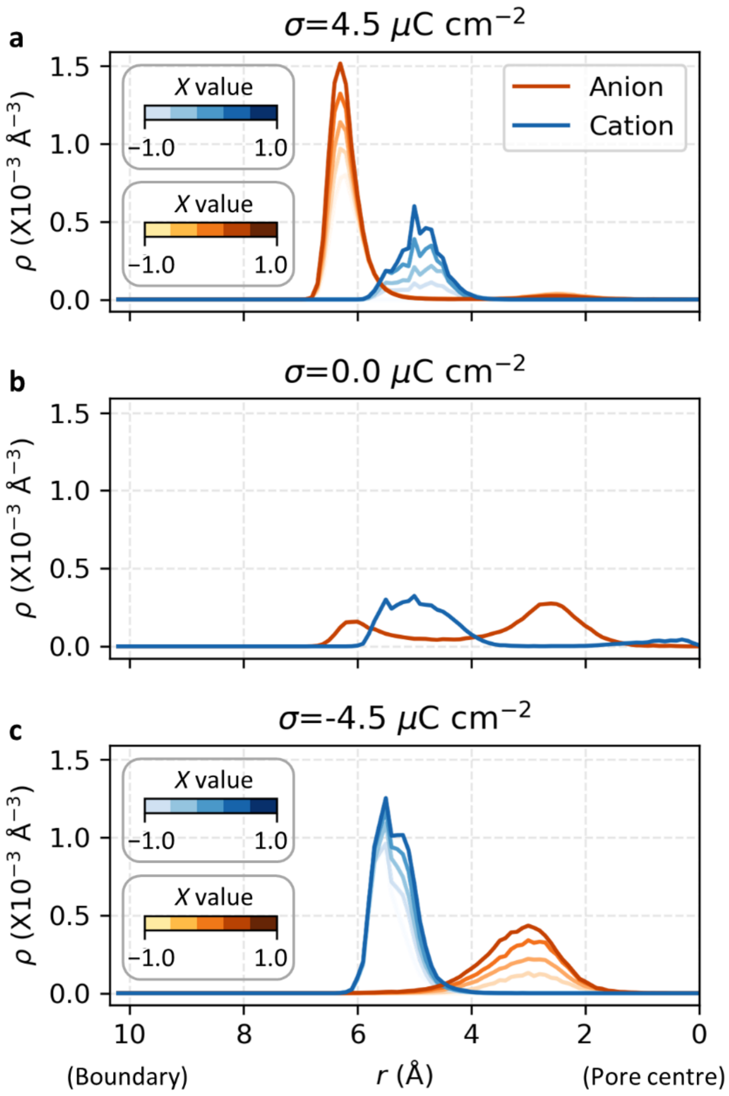

Supplementary Fig. 31. **a–c,** Density (ρ) of cations and anions in $Cu_3(HITP)_2$ are shown with respect to the radial distance (r) from the centre of the MOFs. The saturation of colour indicates the density of components at each X value. It is plotted when surface charge density (σ) is +4.5 $\mu C\ cm^{-2}$ (**a**), σ is 0.0 $\mu C\ cm^{-2}$ (**b**), and σ is −4.5 $\mu C\ cm^{-2}$ (**c**).

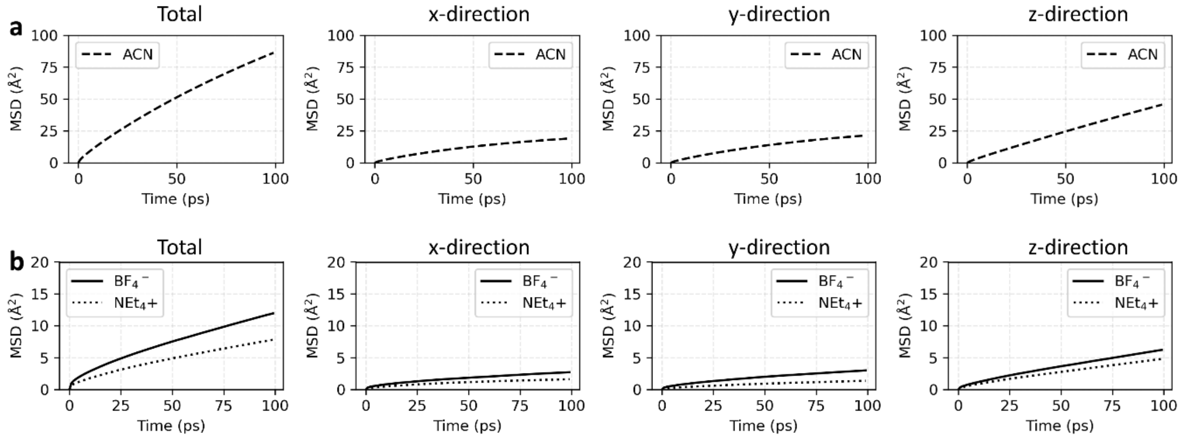

Supplementary Fig. 32. The mean squared displacement (MSD) of the electrolytes at the point of zero charge in the $Cu_3(HITP)_2$ pores. **a,** MSD for the acetonitrile (ACN). **b,** MSD for the ions.

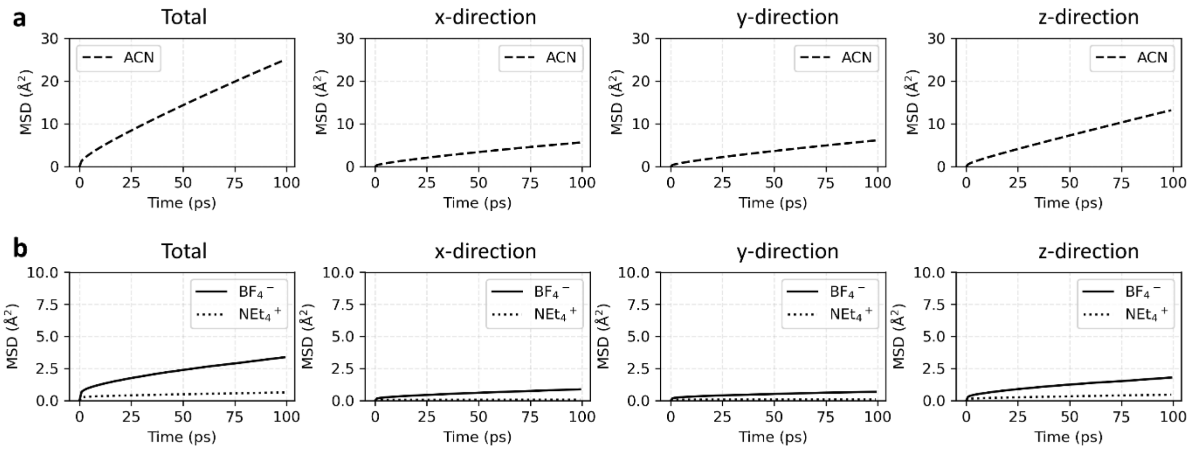

Supplementary Fig. 33. The mean squared displacement (MSD) of the electrolytes at the point of zero charge in the $Cu_3(HHTP)_2$ pores. **a,** MSD for the acetonitrile (ACN). **b,** MSD for the ions.

	Capacitance (μF cm^{-2})		
Current Density / A g^{-1}	Positive	Negative	Switching
0.05	18.5	16.0	19.8
0.1	18.3	15.1	19.1
0.2	18.0	14.2	18.5
0.5	16.5	13.1	17.3

Supplementary Table 1. Areal capacitance values obtained at different current densities from a three-electrode cell (Cell 1), assembled with a $Cu_3(HHTP)_2$ composite working electrode, a YP80F oversized counter electrode, a Ag *pseudo*-reference electrode, and 1 M NEt_4BF_4 in acetonitrile electrolyte (**Supplementary Fig. 8**). All areal capacitance values were calculated from galvanostatic charge-discharge plots. The areal capacitance was measured when charging to the positive potential limit (+0.5 V *vs*. OCV), to the negative potential limit (−0.5 V *vs*. OCV), and across the full potential window (from +0.5 V to −0.5 V; denoted as "Switching").

QM/MM simulation		Capacitance (F g^{-1})	
X value	Charging mechanism	$-4.5 < \sigma < 0$ μC cm^{-2}	$0 < \sigma < 4.5$ μC cm^{-2}
1.0	counterion insertion	231	103
0.5		154	154
0.0	ion-exchange	103	154
−0.5		90	141
−1.0	co-ion removal	77	308

Experiment	Capacitance (F g^{-1})		
Potential / V *vs.* OCV	Negative	Positive	Switching
0.5*	97.7	82.1	104.0

*average of two independent measurements

Supplementary Table 2. Gravimetric capacitance values from the QM/MM simulation. σ is surface charge density. It is calculated by dividing the areal capacitance (**Table 1** in the main text) by the surface area of the supercell (**Supplementary Fig. 1**). The experimental range is calculated from galvanostatic charge-discharge curves by charging to positive potentials *vs.* OCV, to negative potentials *vs.* OCV, and between both positive and negative potentials of the same magnitude denoted as "Switching". "Positive", "Negative", and "Switching" means the potential ranges from 0.5 V to 0 V, −0.5 V to 0 V, and +0.5 V to −0.5 V, respectively. The data were collected between ±0.5 V *vs.* OCV , which is the stable potential window of this MOF, and at a current density 0.05 A g^{-1} to limit kinetic effects. The experiments generally produce lower gravimetric capacitance values than theoretically predicted. This is likely due to the fact that the theoretically predicted surface area is much greater than the experimentally determined value for $Cu_3(HHTP)_2$ in the electrode film (1286 m^2 g^{-1} *vs.* 551m^2 g^{-1}). This may be due to imperfect crystallinity of the MOF or the blockage of some pores by the PTFE binder in the electrodes, both of which may lead to the presence of inaccessible surface area in the experimental $Cu_3(HHTP)_2$ electrodes. Thus, areal capacitance, normalised by the BET surface area, is appropriate for comparing experimental capacitance with simulated values.

Supplementary Note 1. Potential drop from the solvent molecules

The solvent molecules align at the electrochemical interface because of their interaction with the electrode. This causes an interfacial potential drop.[1–4] In the QM/MM simulation, the charge density of all electrolyte components is mapped onto the real-space grid.[5] Its contribution to the total potential drop in the pore is quantified after getting the averaged alignment to the electrode surface and the density of molecules with respect to the radial distance (r) from the centre of the pore.

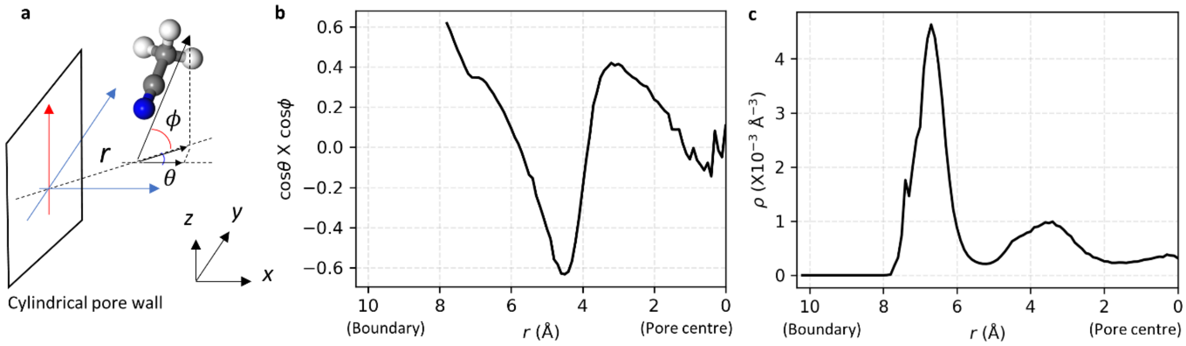

Supplementary Note Fig. 1. a, A schematic figure to define the molecular orientation where the molecular vector is defined along the N-C bond. Two angles are defined where the ϕ is an angle between the acetonitrile and the xy plane. The θ is the angle between the projected vector on the xy plane and the surface normal vector of the cylindrical pore wall. **b,** The $\cos\phi \times \cos\theta$ is used as the order parameter and it is plotted with respect to the radial distance (r) in the cylinder. **c,** Solvent density (ρ) profile with respect to the r in the cylinder. The N atom of the acetonitrile is averaged to calculate the ρ.

The alignment of the solvent molecules is measured by defining the molecular vector of solvent molecule along the N-C bond (N→C direction). Then, two angles are defined where the ϕ is an angle between the acetonitrile and the xy plane, and θ is the angle between the projected vector on the xy plane and the surface normal vector of the cylindrical pore wall (**Supplementary Note Fig. 1a**). The $\cos\phi \times \cos\theta$ is used as the order parameter and it is plotted with respect to the r (**Supplementary Note Fig. 1b**). The density of the solvent is measured by

counting the nitrogen atom of the acetonitrile, and then it is averaged with respect to the *r* from the pore centre (**Supplementary Note Fig. 1c**).

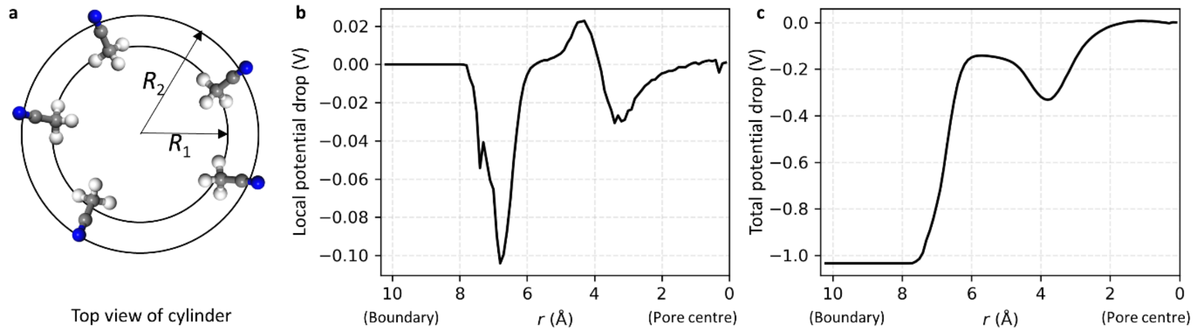

Supplementary Note Fig. 2. a, Schematic figure to approximate the distribution of acetonitrile molecules in the MOFs as the charged cylinders. At certain radial distance (*r*) from the pore centre, a radius of inner cylinder (R_1), and a radius of outer cylinder (R_2) can be defined based on their position. **b,** Local potential drop profiles with respect to the *r* from the solvent. **c,** Total potential drop profiles with respect to the *r* from the solvent.

We approximate the distribution of in-pore acetonitrile molecules as multiple charged cylinders (**Supplementary Note Fig. 2a**). Then, using the radially averaged alignment, density profile, and dipole moment of acetonitrile molecule, the local potential drop at a certain *r* can be calculated based on the Gauss's law (**Supplementary Note Fig. 2b**). The details to get the potential drop in the charged cylinders are explained in the **Supplementary Note 2**. Finally, the total potential drop is achieved by integrating the local potential drop with respect to the *r* (**Supplementary Note Fig. 2c**).

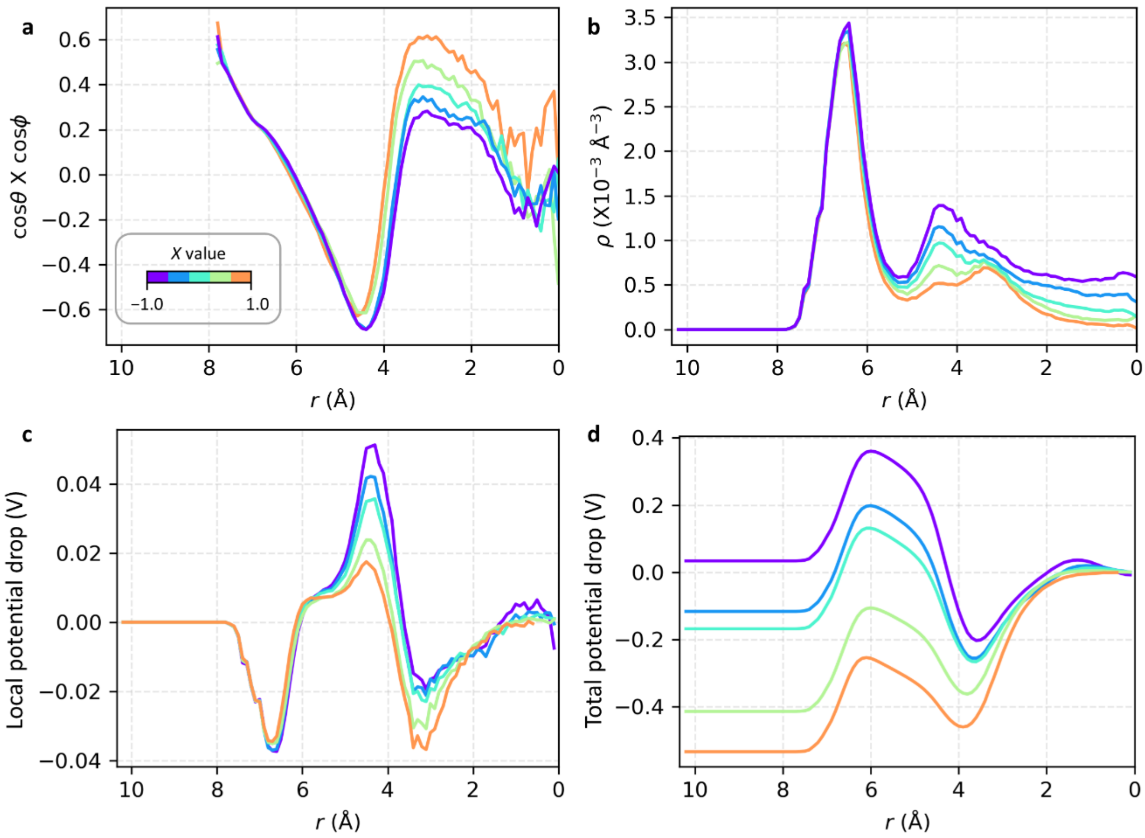

Supplementary Note Fig. 3. The dielectric screening from the acetonitrile molecules when the surface charge density (σ) is −4.5 μC cm^{-2} with respect to the X value. **a,** An averaged molecular orientation with respect to the radial distance (r) in the cylindrical pores. The ϕ is an angle between the acetonitrile and the xy plane. The θ is the angle between the projected vector on the xy plane and the surface normal vector of the cylindrical pore wall. **b,** A number density (ρ) profile with respect to the r. **c,** Local potential drop profiles with respect to the r from the solvent. **d,** Total potential drop profiles with respect to the r from the solvent.

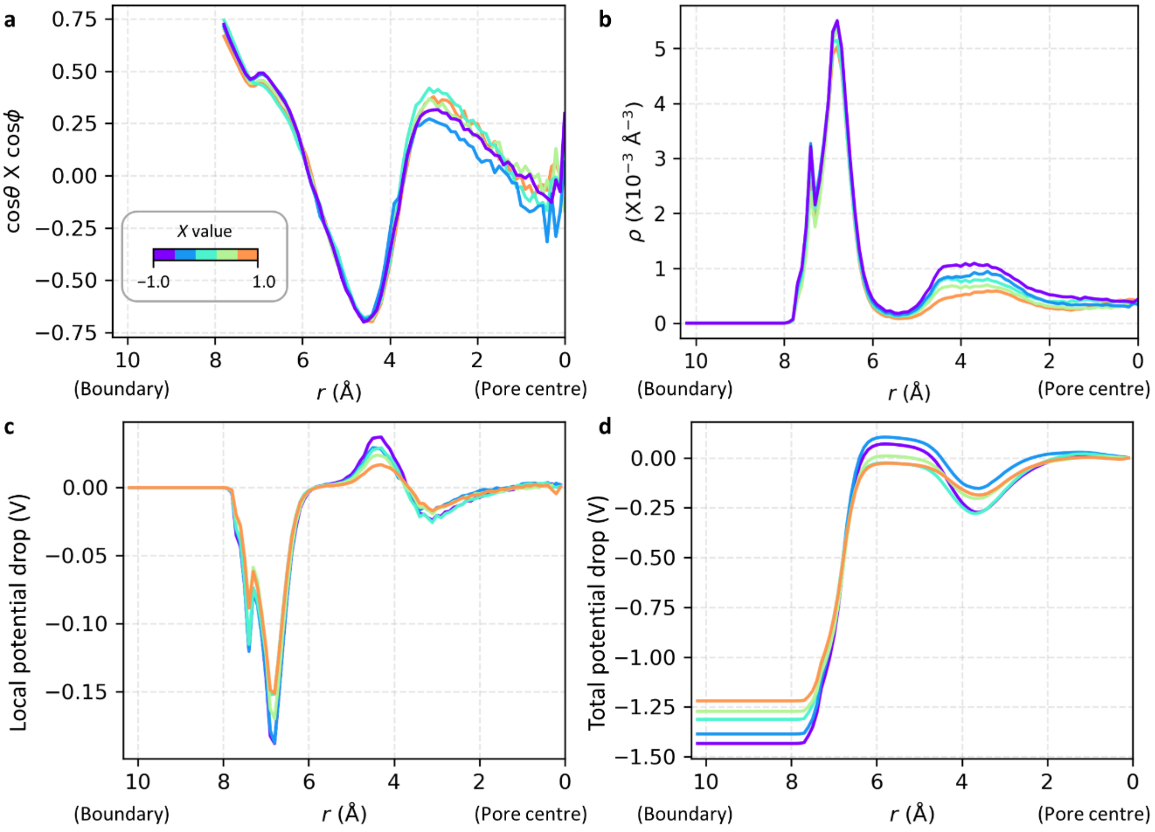

Supplementary Note Fig. 4. The dielectric screening from the acetonitrile molecules when the surface charge density (σ) is +4.5 μC cm^{-2} with respect to the X value. **a,** An averaged molecular orientation with respect to the radial distance (r) in the cylindrical pores. The ϕ is an angle between the acetonitrile and the xy plane. The θ is the angle between the projected vector on the xy plane and the surface normal vector of the cylindrical pore wall. **b,** A number density (ρ) profile with respect to the r. **c,** Local potential drop profiles with respect to the r from the solvent. **d,** Total potential drop profiles with respect to the r from the solvent.

We performed this quantification by varying the X value when σ is −4.5 μC cm^{-2} (**Supplementary Note Fig. 3**) and when σ is +4.5 μC cm^{-2} (**Supplementary Note Fig. 4**). Those data are employed to find the origin of the charging mechanism dependency to the capacitance. It is also employed to analyse the interface of the $Cu_3(HITP)_2$ (**Supplementary Note Fig. 5–7**).

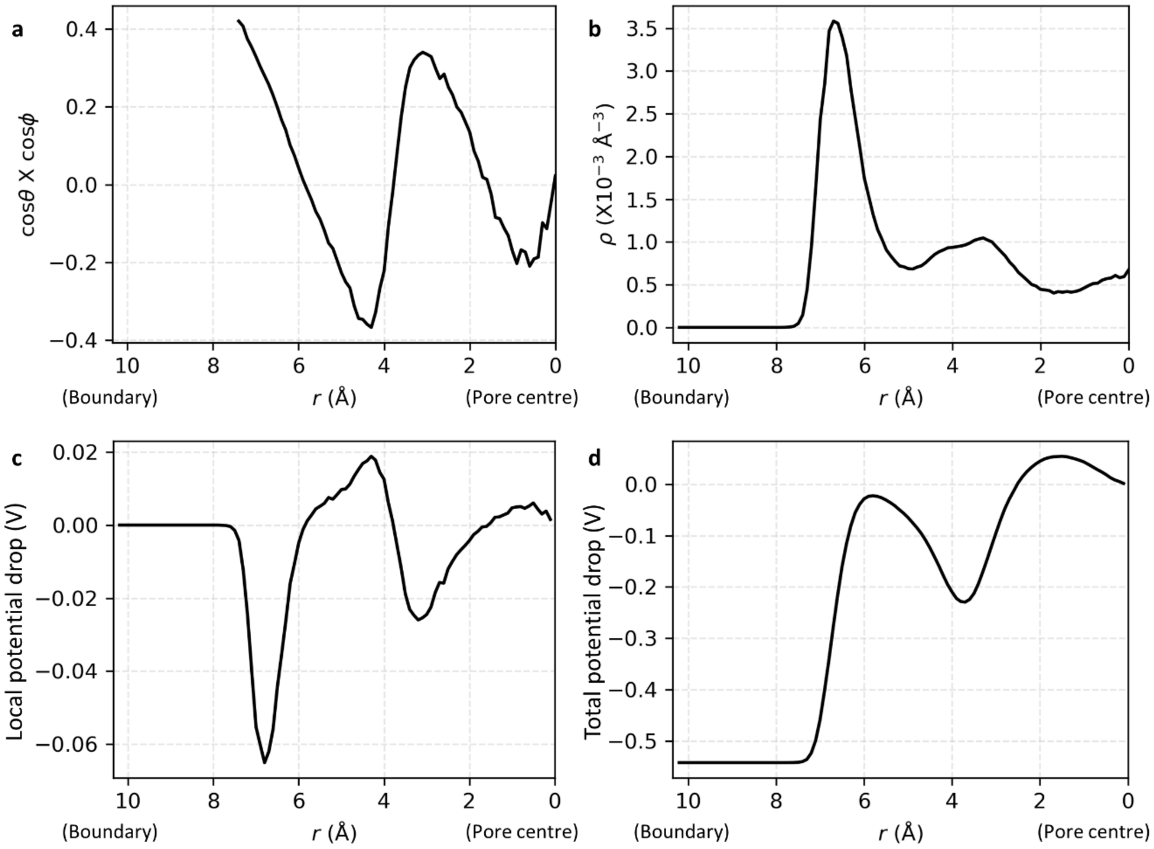

Supplementary Note Fig. 5. The dielectric screening from the acetonitrile molecules at the $Cu_3(HITP)_2$ electrochemical interface when the surface charge density (σ) is 0.0 µC cm^{-2}. **a,** An averaged molecular orientation with respect to the radial distance (r) in the cylindrical pores. The ϕ is an angle between the acetonitrile and the xy plane. The θ is the angle between the projected vector on the xy plane and the surface normal vector of the cylindrical pore wall. **b,** A number density (ρ) profile with respect to the r. **c,** Local potential drop profiles with respect to the r from the solvent. **d,** Total potential drop profiles with respect to the r from the solvent.

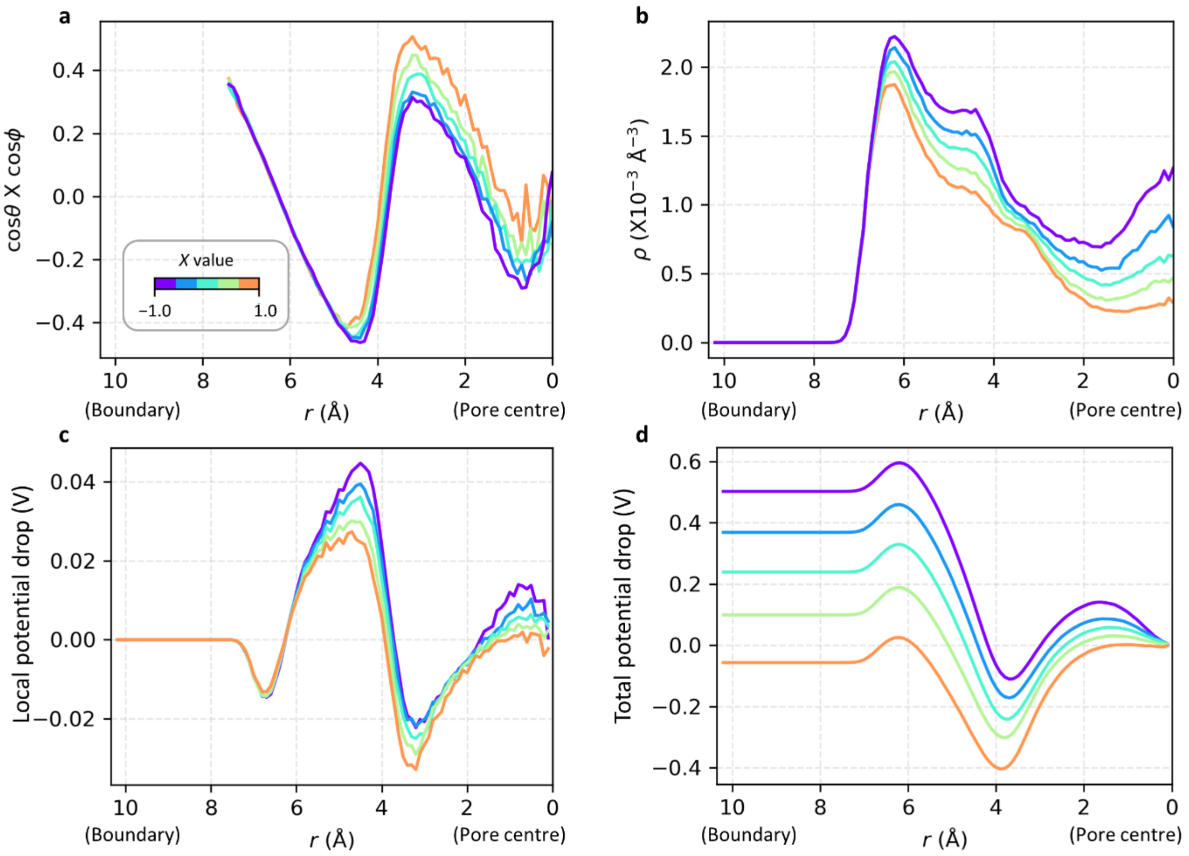

Supplementary Note Fig. 6. The dielectric screening from the acetonitrile molecules when the surface charge density (σ) is −4.5 μC cm^{-2} with respect to the X value at the $Cu_3(HITP)_2$ electrochemical interface. **a,** An averaged molecular orientation with respect to the radial distance (r) in the cylindrical pores. The ϕ is an angle between the acetonitrile and the xy plane. The θ is the angle between the projected vector on the xy plane and the surface normal vector of the cylindrical pore wall. **b,** A number density (ρ) profile with respect to the r. **c,** Local potential drop profiles with respect to the r from the solvent. **d,** Total potential drop profiles with respect to the r from the solvent.

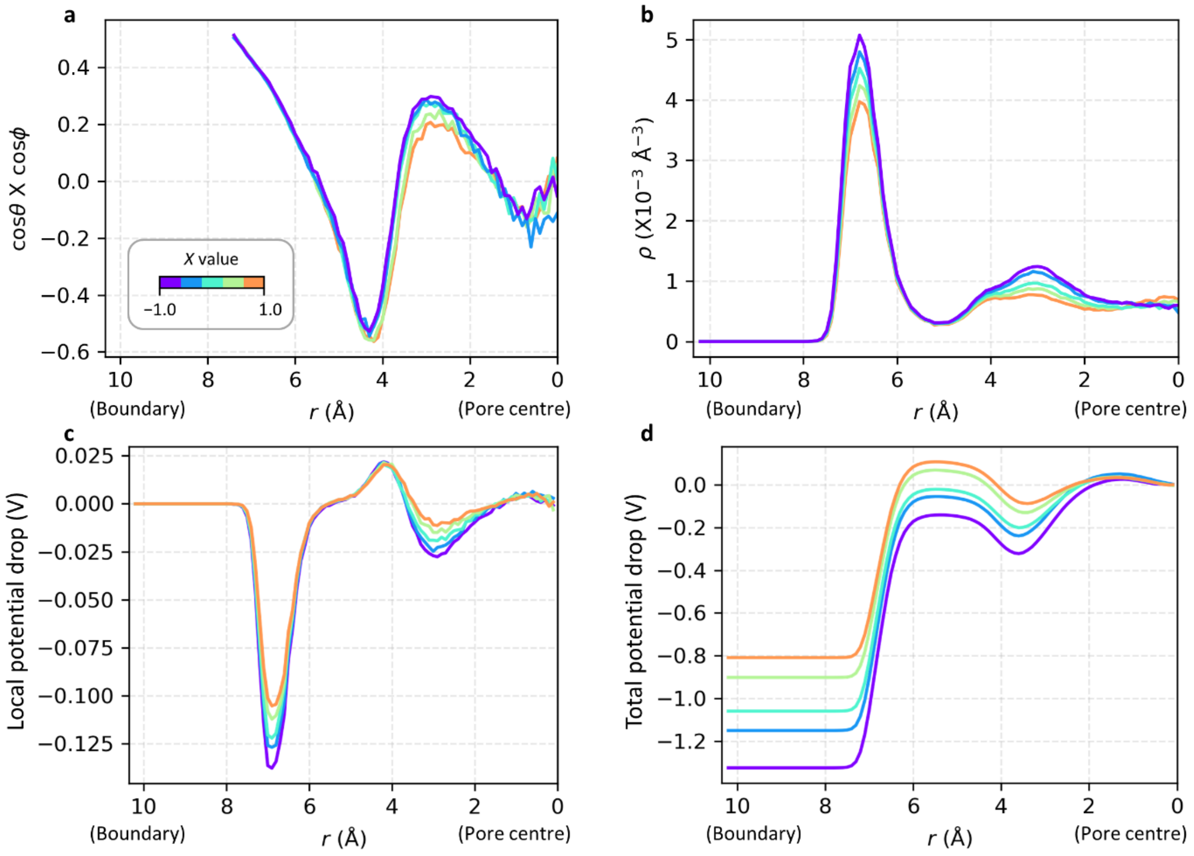

Supplementary Note Fig. 7. The dielectric screening from the acetonitrile molecules when the surface charge density (σ) is +4.5 μC cm^{-2} with respect to the X value at the $Cu_3(HITP)_2$ electrochemical interface. **a,** An averaged molecular orientation with respect to the radial distance (r) in the cylindrical pores. The ϕ is an angle between the acetonitrile and the xy plane. The θ is the angle between the projected vector on the xy plane and the surface normal vector of the cylindrical pore wall. **b,** A number density (ρ) profile with respect to the r. **c,** Local potential drop profiles with respect to the r from the solvent. **d,** Total potential drop profiles with respect to the r from the solvent.

Supplementary Note 2. Potential drop in MOFs

The potential drop in the MOFs is quantified after approximating the distribution of ions as multiple charged cylinders (**Supplementary Note Fig. 8**) and allowing their trajectory to vary over sufficient time. Then, the potential drop (ΔV) between charged cylinder can be calculated based on the Gauss's law as

$$\Delta V = \frac{Q}{2\pi\epsilon_0 l}\int_{R_1}^{R_2}\frac{dr}{r} = \frac{Q}{2\pi\epsilon_0 l}\ln r\,|_{R_1}^{R_2}$$

where, r is the radial distance from the pore centre, R_1 is a radius of inner cylinder, R_2 is a radius of outer cylinder, Q is amount of charge accumulated in the cylinder, l is the height of cylinder, and ϵ_0 is vacuum permittivity. Here, we quantify the Q, R_1, and R_2 based on the radially averaged distribution (**Supplementary Fig. 11**).

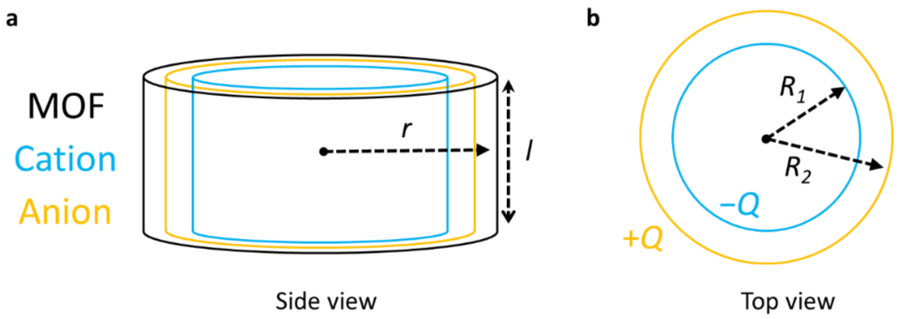

Supplementary Note Fig. 8. a,b, Schematic figure to approximate the distribution of ions in the MOFs as the charged cylinder viewing at side view (a) and top view (b).

This makes it possible to analyse the total potential drop by decomposing it into multiple potential drops in the pores. When σ is 0.0 μC cm^{-2}, there are three charged cylinders, which make two potential drops (**Supplementary Note Fig.9a**), and their summation is almost zero (**Supplementary Note Fig. 9b**). When σ is +4.5 μC cm^{-2}, the analysis is performed varying the X value. There exists one more charged cylinder, which is the positively charged

MOF, and there exist three potential drops (**Supplementary Note Fig. 10a**), and their summated magnitude gives non-monotonically increasing behaviour (**Supplementary Note Fig. 10b**), consistent to non-monotonically decreasing behaviour of capacitance from the QM/MM simulation.

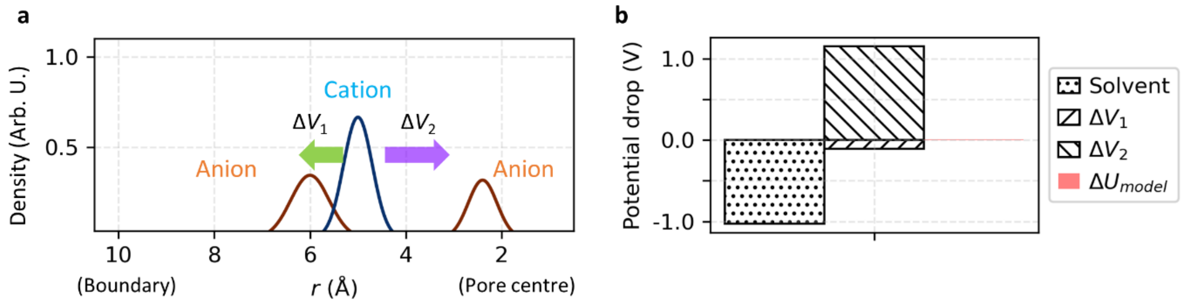

Supplementary Note Fig. 9. a, The density of cations and anions in the MOF is illustrated with respect to the radial distance (r) from the centre of the MOF at point of zero charge (PZC). It is based on the **Supplementary Fig. 19**. The two major electric fields between the different components are shown with coloured arrows named ΔV_1 and ΔV_2. **b,** The potential drop inside the MOF is shown using cylindrical capacitor models. The total potential drop (ΔU_{model}) is decomposed into three terms, where two of them are ΔV_1 and ΔV_2. The potential drop due to the effective dielectric screening from the solvent molecules (Solvent) is also shown.

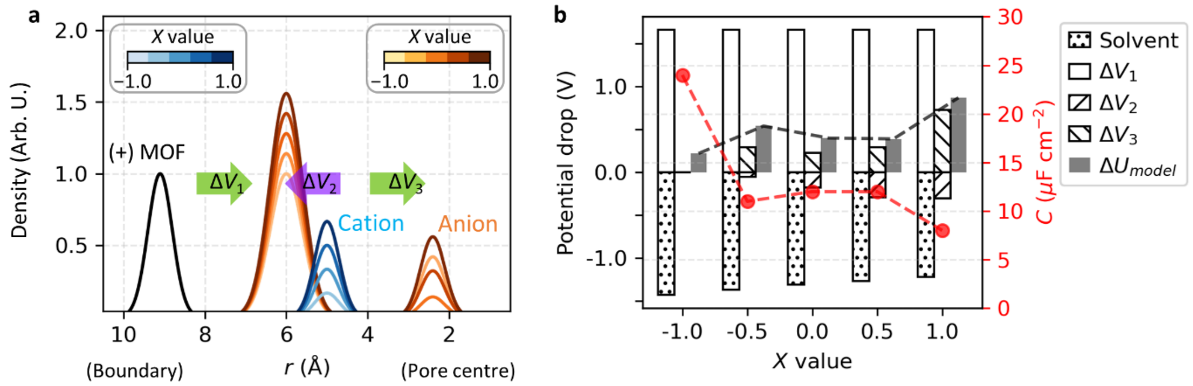

Supplementary Note Fig. 10. a, The density of excess charges in the MOF are illustrated with respect to the radial distance (r) from the centre of the MOF, when the surface charge density (σ) is +4.5 μC cm^{-2}. It is based on the **Supplementary Fig. 19**. The saturation of colour indicates the density of components at each X value. The three major electric fields between the different components are shown with coloured arrows named ΔV_1, ΔV_2, and ΔV_3. **b,** The potential drop inside the MOF is shown with respect to the X value using cylindrical capacitor models. The total potential drop (ΔU_{model}) is decomposed into four terms, where three of them are ΔV_1, ΔV_2, and ΔV_3. The potential drop due to the effective dielectric screening from the

solvent molecules (Solvent) is also shown. Differential capacitance (C) value from the QM/MM simulation is plotted together.

It is also employed to analyse the interface of $Cu_3(HITP)_2$ (**Supplementary Note Fig. 11–12**). When σ is 0.0 μC cm^{-2}, there are three charged cylinders, which make two potential drops (**Supplementary Note Fig. 11a**), and their summation is almost zero (**Supplementary Note Fig. 11b**).

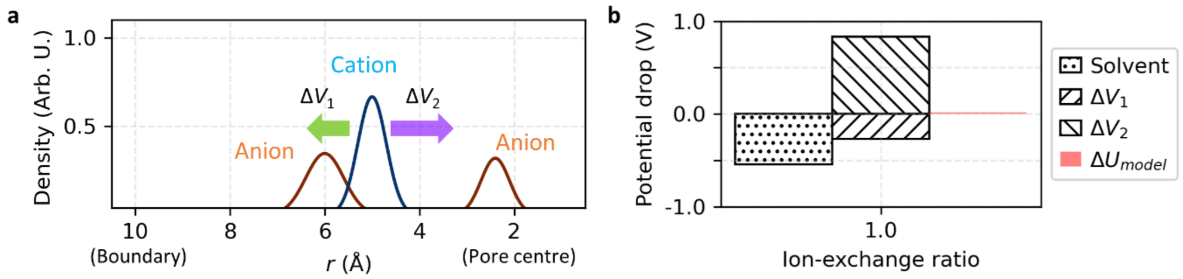

Supplementary Note Fig. 11. a, The density of cations and anions in the MOF is illustrated with respect to the radial distance (r) from the centre of the $Cu_3(HITP)_2$ MOF at PZC. It is based on the **Supplementary Fig. 31**. The two major electric fields between the different components are shown with coloured arrows named ΔV_1 and ΔV_2. **b,** The potential drop inside the MOF is shown using cylindrical capacitor models. The total potential drop (ΔU_{model}) is decomposed into three terms, where two of them are ΔV_1 and ΔV_2. The potential drop due to the effective dielectric screening from the solvent molecules (Solvent) is also shown.

When σ is ±4.5 μC cm^{-2}, the analysis is performed varying the X value. There exist two potential drops (**Supplementary Note Fig. 12a** and **Supplementary Note Fig. 12c**), and their summated magnitude gives monotonically decreasing or constant behaviour (**Supplementary Note Fig. 12b** and **Supplementary Note Fig. 12d**), consistent to monotonically increasing or constant behaviour of capacitance from the QM/MM simulation.

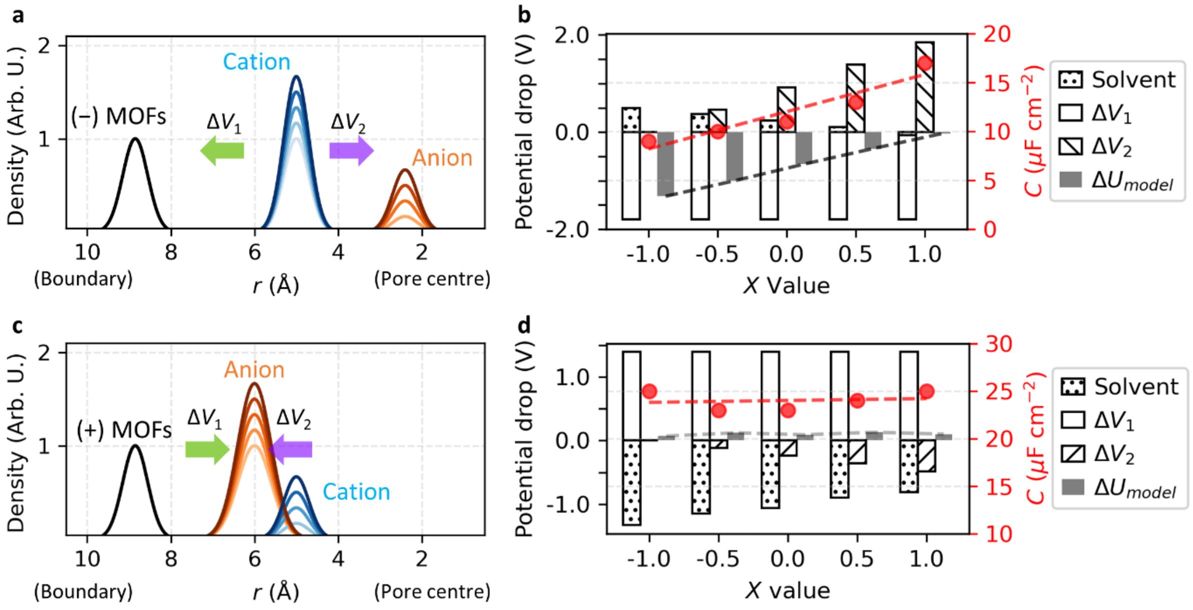

Supplementary Note Fig. 12. a,c, The density of excess charges in $Cu_3(HITP)_2$ MOF are illustrated with respect to the radial distance (r) from the centre of the MOFs. It is based on the **Supplementary Fig. 31**. The saturation of colour indicates the density of components at each X value. The surface charge density (σ) is −4.5 μC cm^{-2} (**a**), and +4.5 μC cm^{-2} (**c**). **b,d,** The potential drop inside the MOFs is shown with respect to the X value using cylindrical capacitor models. σ is −4.5 μC cm^{-2} (**b**), and +4.5 μC cm^{-2} (**d**). Differential capacitance (C) value from the QM/MM simulation is plotted together.

Supplementary Reference

(1) Shin, S.-J.; Kim, D. H.; Bae, G.; Ringe, S.; Choi, H.; Lim, H.-K.; Choi, C. H.; Kim, H. On the Importance of the Electric Double Layer Structure in Aqueous Electrocatalysis. *Nat. Commun.* **2022**, *13* (1), 174. https://doi.org/10.1038/s41467-021-27909-x.
(2) Grahame, D. C. The Electrical Double Layer and the Theory of Electrocapillarity. *Chem. Rev.* **1947**, *41* (3), 441–501. https://doi.org/10.1021/cr60130a002.
(3) Bockris, J. O.; Devanathan, M. A. V.; Muller, K. On the Structure of Charged Interfaces. *Proc. R. Soc. A* **1963**, *274* (1356), 55–79. https://doi.org/10.1098/rspa.1963.0114.
(4) Sugahara, A.; Ando, Y.; Kajiyama, S.; Yazawa, K.; Gotoh, K.; Otani, M.; Okubo, M.; Yamada, A. Negative Dielectric Constant of Water Confined in Nanosheets. *Nat. Commun.* **2019**, *10* (1), 850. https://doi.org/10.1038/s41467-019-08789-8.
(5) Lim, H.-K.; Lee, H.; Kim, H. A Seamless Grid-Based Interface for Mean-Field QM/MM Coupled with Efficient Solvation Free Energy Calculations. *J. Chem. Theory Comput.* **2016**, *12* (10), 5088–5099. https://doi.org/10.1021/acs.jctc.6b00469.
